# Under What Circumstances Do Wood Products from Native Forests Benefit Climate Change Mitigation?

**DOI:** 10.1371/journal.pone.0139640

**Published:** 2015-10-05

**Authors:** Heather Keith, David Lindenmayer, Andrew Macintosh, Brendan Mackey

**Affiliations:** 1 Fenner School of Environment and Society, The Australian National University, Canberra, ACT, Australia; 2 Centre for Climate Law and Policy, ANU College of Law, The Australian National University, Canberra ACT, Australia; 3 Griffith Climate Change Response Program, Griffith University, Southport, Queensland, Australia; University of New South Wales, AUSTRALIA

## Abstract

Climate change mitigation benefits from the land sector are not being fully realised because of uncertainty and controversy about the role of native forest management. The dominant policy view, as stated in the IPCC’s Fifth Assessment Report, is that sustainable forest harvesting yielding wood products, generates the largest mitigation benefit. We demonstrate that changing native forest management from commercial harvesting to conservation can make an important contribution to mitigation. Conservation of native forests results in an immediate and substantial reduction in net emissions relative to a reference case of commercial harvesting. We calibrated models to simulate scenarios of native forest management for two Australian case studies: mixed-eucalypt in New South Wales and Mountain Ash in Victoria. Carbon stocks in the harvested forest included forest biomass, wood and paper products, waste in landfill, and bioenergy that substituted for fossil fuel energy. The conservation forest included forest biomass, and subtracted stocks for the foregone products that were substituted by non-wood products or plantation products. Total carbon stocks were lower in harvested forest than in conservation forest in both case studies over the 100-year simulation period. We tested a range of potential parameter values reported in the literature: none could increase the combined carbon stock in products, slash, landfill and substitution sufficiently to exceed the increase in carbon stock due to changing management of native forest to conservation. The key parameters determining carbon stock change under different forest management scenarios are those affecting accumulation of carbon in forest biomass, rather than parameters affecting transfers among wood products. This analysis helps prioritise mitigation activities to focus on maximising forest biomass. International forest-related policies, including negotiations under the UNFCCC, have failed to recognize fully the mitigation value of native forest conservation. Our analyses provide evidence for decision-making about the circumstances under which forest management provides mitigation benefits.

## Introduction

Storage of carbon as biomass in the land sector is an important activity for climate change mitigation. Loss of carbon from deforestation and degradation has contributed 35% of the accumulated anthropogenic carbon dioxide concentration in the atmosphere [1], and annually is around 10% of global anthropogenic emissions [2]. The global amount of emissions from deforestation and degradation continues to increase, but the proportional contribution to total emissions has decreased during the 20^th^ century with increased fossil fuel use. The cross-over point of these sources of emissions is estimated to have occurred either early or late in the 20^th^ century, depending on whether decomposition of wood products and changes in soil organic carbon due to land-use are accounted for [3]. In Australia, an estimated 44% of the carbon stock in temperate forests has been emitted due to deforestation [4].

Reducing these emissions and restoring the land carbon stock by identifying strategies for forest management that increase carbon storage is an important component of a comprehensive approach to climate change mitigation [5]. Carbon is stored in forest biomass, wood products and waste material in landfill. Furthermore, it is argued that mitigation benefits can be derived from using forest biomass as a feedstock for bioenergy, substituting for an equivalent amount of fossil fuel energy, and avoiding the associated carbon dioxide emissions. Similarly, it is claimed that use of wood products with lower embodied energy than other construction products can avoid emissions. Given that a range of forest management and carbon accounting strategies is possible with varying mitigation outcomes, a critical question is: how can we best manage forests and their harvested products to maximise carbon storage in the land sector and minimise net anthropogenic greenhouse gas emissions?

We focus this study on native forests, that is, self-regenerating ecosystems where ecological processes dominate. This is because their management for competing resources is controversial and scientific evidence is needed to evaluate options [5]. The options for carbon storage are often depicted as a dichotomy between commercial harvesting and conservation, although a range of management strategies exist for native forests. With commercial harvesting, native forests are logged at regular periods and the woody biomass is used as the raw materials for manufactured wood and paper products. Mitigation benefits potentially can arise from the carbon stored in the wood products, the forest regrowth that occurs in between harvests, and avoided fossil fuel emissions due to substitution by wood products and bioenergy. A strategy of conservation, whereby native forests are not harvested, allows carbon stocks to reflect ecosystem processes of growth, mortality, decomposition, and self-regeneration. A benefit of the native forest conservation strategy is that the forest carbon stock is at a maximum given the environmental conditions and disturbance regimes that characterise the landscape. Additionally, stability of these natural carbon stocks is conferred by the capacity for resilience and self-regeneration of natural ecosystem processes [6]. Under both strategies, fluctuations in the native forest carbon stock occur due to natural disturbances, especially wildfire, storms, pests and diseases [7].

Assessing the relative mitigation benefit of these two native forest management strategies depends on the amount of carbon transferred between natural and manufactured stocks and the longevity of these stocks. The key calculation is the impact a forest management strategy has on net land and fossil carbon stocks, which are then reflected in the atmospheric carbon dioxide concentration.

Different conclusions about mitigation benefits of forest management strategies have been reported in the literature over the last two decades [7–15] (Table A in S1 Appendix). The view stated in the IPCC’s Fifth Assessment Report is that sustainable harvesting yielding wood products generates the largest mitigation benefit [16]. This opinion has been advocated by politicians responsible for national forest policy [17], national forest policy statements [18], state agencies responsible for managing publicly-owned native forest land [19,20], and communicated to the public in media campaigns [21]. However, the published literature shows that the circumstances under which forest management provides mitigation benefits varies and is not universal. This uncertainty in, and controversy about, the role of native forests in climate change mitigation has led to inconsistencies in national and international policies about land management and climate policy.

Many of the differences in previous studies’ conclusions are due to inconsistencies between them concerning: (i) the forest conditions in the reference case; (ii) the temporal scales over which the analyses are undertaken; (iii) the spatial scale of the analyses; and (iv) the substitution assumptions. The forest condition in the reference case (what would occur in the counterfactual if there was no change in management practice) determines the basis for comparison of carbon stocks against which potential gains and losses can be assessed. An initial loss of carbon occurs when a native forest, in an old-growth or primary condition, is harvested and replaced by a forest managed for products, or regrowth forest [8,22]. The difference in carbon stock can be assessed from the native forest as an average within its natural disturbance regime, compared with the managed forest as an average over the rotation period [23]. Temporal scales relevant for effective climate mitigation activities are within the next few decades [24]. A temporal imbalance between carbon emissions and uptake rates in the order of many decades may not provide a net mitigation benefit [25,26]. The assumptions underlying the mitigation benefits of product or energy substitution are often not made explicit and may not account for all factors. In our analyses, we have considered these issues, tested a range of parameter values, and explicitly defined the system conditions.

Our objective was to assess native forest management strategies to determine the circumstances that provide maximum benefits for climate change mitigation. We simulated the effect on carbon stocks of management strategies for two native forest systems from Australia: (i) mixed eucalypt forest in the South Coast Region of New South Wales (NSW); and (ii) Mountain Ash forest in the Central Highlands Region of Victoria. For both case studies, we compared the carbon stocks under two contrasting management scenarios: (i) harvested native forests, with options for accounting for the carbon storage in regrowth forest biomass, wood and paper products, landfill, and the carbon benefits of bioenergy substituted for fossil fuel energy, and (ii) conserved native forests, accounting for carbon storage in forest biomass, with options for accounting for substitution by non-native wood products. We tested a range of parameter values describing carbon stock dynamics that are general for other forest systems and identified key issues for analysing carbon stock changes that drive the outcomes of accounts.

The context of our research was to contribute to advancing the scientific understanding of carbon stock dynamics in forest system. This information is also relevant for the decision-making process about the relative benefits of native forest management strategies for climate change mitigation. The management of public native forests is determined by public policy through the political process. Decisions about forest management must account for environmental, social and economic factors. Simulations from scenarios of different forest management strategies are based on a range of input data and assumptions, which may be limited in some respects but represent the best currently available. These simulations demonstrate some of the consequences of different actions and so contribute information to the public, policy-makers and politicians.

## Methods

### Defining the carbon accounting framework

**Fig 1:**
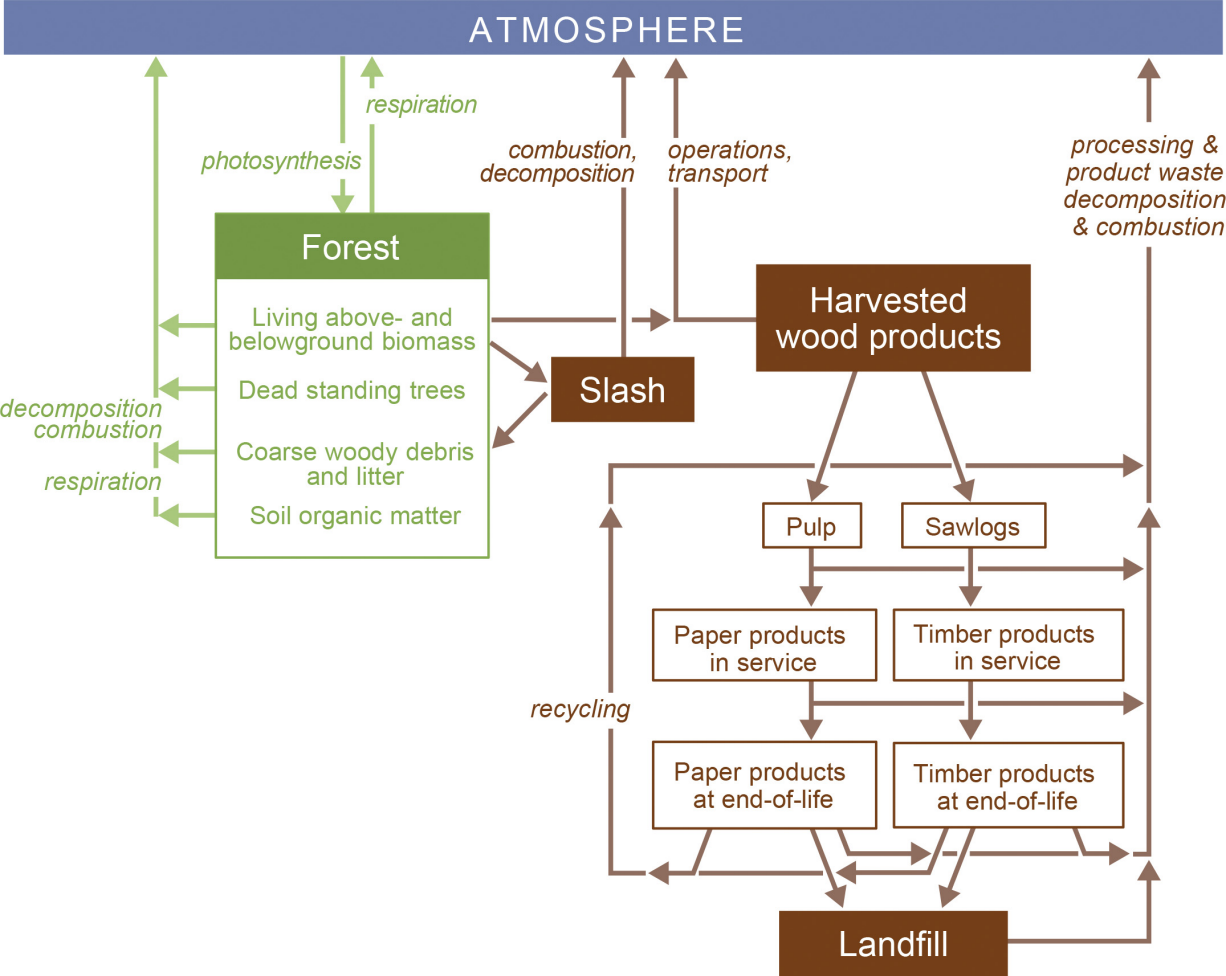
Carbon stocks and transfers in a forest and harvested wood products system. Boxes represent stocks of carbon, and arrows represent transfers between stocks with the process defined in italics.

Our analyses required accounting for all carbon stocks in the native forest management system and post-harvest products: forest biomass carbon; harvested wood products; waste material; and potential substitution of wood products for other materials, and bioenergy for fossil fuel as a source of energy. All components of biomass were included in the carbon stock: living and dead, above-and below-ground biomass. Owing to data limitations, soil carbon stocks were excluded (Australia does not account for soil carbon in harvested native forests in its national greenhouse gas accounts for the same reason [27]). Changes in carbon stocks were described by transfers of biomass through forest harvesting, forest regrowth, wood products processing and use, rates of decomposition and combustion, energy conversion efficiencies, and disposal of waste (Fig 1). The proportions of each of these stocks vary depending on the forest type and species, silvicultural treatment and market for products. Carbon stocks were defined by their magnitude, longevity and stability [28].

We selected two native forest management systems in Australia for our investigations that differed in forest ecosystem type, geographic location, forest management regimes, and the wood products derived from the timber harvesting. These forest types provide a significant proportion of harvested wood volumes in each state [29] and the forest management activities in these regions cover a range of logging intensities, which are comparable with forest management practices in other temperate forests.

We used a simulation model to describe the stocks of carbon, transfers between stocks and resulting changes in stocks for these forest systems. The main parameters in the model of the harvested forest system are listed in Table 1, together with the input data used to calibrate the models for the two case study regions. Data values were sourced from the most relevant and reliable published sources. Details of the derivation of data and range in values from different sources are described in S2 Appendix.

**Table 1:** Parameters describing carbon stocks and stock changes in the case study forest systems.

Parameter	South Coast NSW	ref	Mountain Ash Victoria	ref
Average carbon stock in total biomass across the forest region (tC ha^-1^)(Reference case)	116	[13]	485	[30]
Maximum carbon stock in aboveground living biomass at	130 _(a)_	[13]	775	[30]
a forest site (tC ha^-1^)	250 _(b)_	S2		
Biomass accumulation rate (tC ha^-1^)	AGB = 130 * (1 –exp(-0.022 * age))^^0.52^ _(a)_	S2	TB = 1200 * (1 –exp(-0.0045 * age)) ^^0.7^	[30]
	AGB = 250 * (1 –exp(-0.003 * age))^^0.45^ _(b)_	S2	AGB = 620 * (1 –exp(-0.0065 * age)) ^^0.75^	[31]
Mean annual increment (tC ha^-1^ yr^-1^)	1.64	*	6.45	*
MAI for species (m^3^ ha^-1^ yr^-1^)	4.1	[32]	14	[32]
Increment converted from MAI (tC ha^-1^ yr^-1^)	1.44		5.9	
Proportion of aboveground biomass removed off-site as	0.35 _(a)_	[35]	0.4	[33]
wood products	0.22 _(b)_	[37]		
	0.61 _(b)_	[37]		
Biomass removed off-site (tC ha^-1^ yr^-1^)	0.36	*	1.58	*
Decomposition rate of slash and coarse woody debris	0.0486 yr^-1^	[34]	0.0486 yr^-1^	[34]
Proportion of wood products used for sawlogs	0.38	[13]	0.275	[33]
Production of sawlog products in-service (tC ha^-1^ yr^-1^)	0.058		0.158	*
Production of pulp products in-service (tC ha^-1^ yr^-1^)	0.28		0.79	*
Decomposable fraction of wood products in landfill: DOC_f_	0.23	[35]	0.23	[35]
Decay rate in landfill: k	0.004 yr^-1^	[36,29]	0.004 yr^-1^	[36]

AGB aboveground living biomass, TB total living biomass,

* model output, S2 Appendix

^(a)^ Parameter value used in the base case simulation,

^(b)^ range in values used in the sensitivity analysis

### Case study 1: Mixed native eucalypt forest in the South Coast Region, NSW

The South Coast of NSW is a sub-region of Forestry Corporation of NSW’s (FCNSW) commercial native forest estate. The sub-region is centred on Batemans Bay (35°42’26”S, 150°10’38”E) and extends north to Nowra, south to Cobargo and west to Queanbeyan. A diversity of forest types occur with an associated range in productivities, in a complex mosaic pattern with species composition and forest structure related to environmental conditions, particularly soil moisture and nutrient availability [37]. Forest types include Spotted Gum (*Corymbia maculata*), Coastal Moist Forest (*Eucalyptus saligna*, *E*. *pilularis*, *E*. *botryoides*, *Synacarpia glomulifera*), Silvertop Ash (*E*. *sieberi*), Coastal Dry forest (*C*. *gummifera*, *E*. *piperita*, *E*. *acmenoides*, *E*. *umbra*, *E*. *resinifera*, *E*. *paniculata*, *E*. *punctata*, *E*. *longifolia*, *E*. *globoidea*, *E*. *agglomerata*, *E*. *tereticornis*, *Angophora floribunda*, *E*. *moluccana*, *E*. *crebra*, *E*. *fibrosa*, *E*. *rudderi*), Brown Barrel (*E*. *fastigata*), Yellow Stringybark and Gum (*E*. *muellerana*, *E*. *melliodora*), and Tableland Gum (*E*. *dalrympleana*, *E*. *viminalis*) [38–40]. The South Coast sub-region produces approximately 20% of the wood volume from native forests in NSW [41].

The native forest management regimes applied in the region since the mid-1900s have covered a range of harvesting intensities: low intensity treatments such as single tree selective harvesting through to higher intensity group selection harvesting [42]. These treatments involve a proportion of the trees being felled either to supply wood products or to improve regeneration capacity of commercially desirable species. These forests are naturally multi-aged as the dominant trees species are not necessarily killed by even severe fire events. For our model, areas logged were defined in three ways: (i) the gross area of the total sub-region that is subject to harvesting; (ii) the net harvestable area which is the area of land where selective harvesting can occur based on the forestry regulations; and (iii) the logged area which is the actual area within which harvesting occurs (Table C in S2 Appendix). Simulations of carbon stock change were analysed for each of these areas to define the proportions of biomass remaining on-site and removed in harvested wood products.

The input data for our model are shown in Tables A, D and E in S2 Appendix. We have used data from FCNSW [9], and have compared these data with the range of data available from other sources. This comparison demonstrates the effect of differences in assumptions in carbon accounting on the outcome of net carbon stock change.

### Case study 2: Mountain Ash native forest in the Central Highlands, Victoria

Our study region in the Central Highlands is between the towns of Marysville, Healesville, Warburton, Neerim South and Rawson in southern Victoria (37°20’–38°0’S and 145°30’–146°20’E) and includes parts of the VicForests Forest Management Areas of Central, Dandenong and Central Gippsland. Native forests in the region consist of several forest types, but we have confined our analysis to the Mountain Ash forest type (predominantly *Eucalyptus regnans*, with some *E*. *delegatensis* and *E*. *nitens*). This forest type currently occurs in State Forests where it is used for commercial wood production, and in conservation reserves and water catchments, some of which are protected. These forests have been harvested for over a century, initially by selective logging but increasingly intensified. Current harvesting practice is clearfelling and slash burning [43–45]. Clearfelling has also included salvage logging after wildfires [46]. The Ash forest type produces approximately 65% of the wood volume from native forests in Victoria [47]. These forests are subject to a disturbance regime of infrequent wildfires [48]. Within the boundary of a wildfire, usually less than half the trees are killed resulting in a mosaic of even-aged and multi-aged forests [49,50].

### Potential Substitution

We included in our simulations estimates of the potential additional mitigation benefits from three forms of substitution. First, substituting biomass as a feedstock for bioenergy to displace fossil fuel-based energy generation. Second, the use of wood products that are assumed to displace more fossil fuel-intensive products. Third, substituting plantation-sourced wood products to displace native forest-sourced wood products, either from new or existing areas of plantations. Plantations are considered as agricultural systems with crops of trees and where human management dominates. Although there can be a continuum of forest management systems, in Australia, native forest and plantation systems are distinct [18,27,51]. Substitution with plantation products provides a mitigation benefit when wood products are produced more efficiently with lower net carbon emissions.

The mitigation benefit of substituting wood products to directly displace fossil fuel energy or indirectly displace more energy-intensive products is defined by displacement factors with units of tC avoided emissions / tC in wood product. This unit represents the net amount of fossil fuel carbon not emitted as the result of 1 tC in biomass used for energy or stored in wood products [12,23]. The displacement factor includes reductions in emissions due to less embodied energy from acquisition of raw materials, transport and processing; avoiding emissions from the calcination process in cement production; and use of wood product residues for bioenergy in processing. Details of the calculation of displacement factors and the range of values for different products are given in Table H in S2 Appendix.

### Simulations of carbon accounting under forest management scenarios

*Reference scenario*: We used the current state of native forest management in commercial state forests as the reference scenario for our simulations. Carbon stocks included the regrowth forest biomass, harvested wood products and waste deposited in landfill. Wood products included chips used for pulp and paper production, and sawlogs used for structural timber, floorboards, poles and landscaping. No wood is used for bioenergy.

We then ran simulations for the following two forest management scenarios.

*Scenario (1)*: Management of native forests for maximum production of wood products and bioenergy. This scenario involved the same harvest intensity as the reference scenario but greater utilisation of products by displacing fossil fuel energy rather than accumulating stocks in harvest residues, landfill and pulp. Carbon stocks were accounted for in the forest regrowth between harvesting cycles, the carbon stock accumulated in manufactured wood products, and the avoided carbon emissions resulting from substituting fossil fuel sourced energy with bioenergy. Biomass for energy generation was derived from slash, wood product processing waste, wood products at-end-of-life instead of being deposited in landfill, and wood chips instead of using them for paper production.

*Scenario (2)*: Management for native forest conservation. In this scenario, we simulated the carbon stocks that would accumulate if the current native forest was protected from harvesting and allowed to function under natural conditions.

To facilitate comparisons between scenarios, in scenario (2) we accounted for the carbon in wood products that were not produced from the native forest and were substituted with other products. We simulated three types of substitution. The first type of substitution used non-wood products that were mostly more fossil fuel intensive to produce. We used an average displacement factor of 2.1 tC tC^-1^ [23] for the gross wood products which were foregone by not harvesting the forest. This factor incorporated all greenhouse gas emissions from the non-wood products. These emissions due to substitution were subtracted from the carbon stock in the conservation native forest to give the net substitution carbon stock (Scenario (2a)). The second type of substitution used plantation wood products. The area of plantation forest required to produce an equivalent amount of wood products as derived from the current native forest was calculated. For the simulation, the same amount of wood products were produced, only they were produced from the plantation area, and the area of conservation native forest was reduced by an equivalent of the area required for the plantation (Scenario (2b)). The third type of substitution used wood products from existing areas of plantations and maintained the entire area of native forest as conserved for carbon storage (Scenario (2c)).

For all scenarios, the area of land, harvest intensity and amount of wood products were kept consistent.

### Sensitivity analysis

Several parameters contribute to uncertainty in the simulated carbon stock change under the different native forest management scenarios: (i) forest carbon accumulation rate and maximum carbon stock in undisturbed forests; (ii) proportion of biomass removed off-site as harvested wood products; (iii) wood products supply; (iv) longevity of wood products; (v) decay rate in landfill; (vi) differentiation of pools within wood and paper products and landfill with different decay rates; (vii) displacement factors for substitution of products; (viii) displacement factors for substitution of bioenergy; (ix) proportion of harvested biomass used for sawlogs and pulp; (x) proportion of slash combusted; and (xi) rotation length of harvesting. Lack of experimental data, both in general and for specific forest types, limit the capacity for simulation. For each of these eleven parameters, we tested a range of values from the literature to determine their effect on carbon stock changes in either the mixed native forest in NSW or the Mountain Ash forest in Victoria, depending on where uncertainty was greatest in these parameters.

## Results

### Mixed native eucalypt forest on the South Coast, NSW

**Fig 2:**
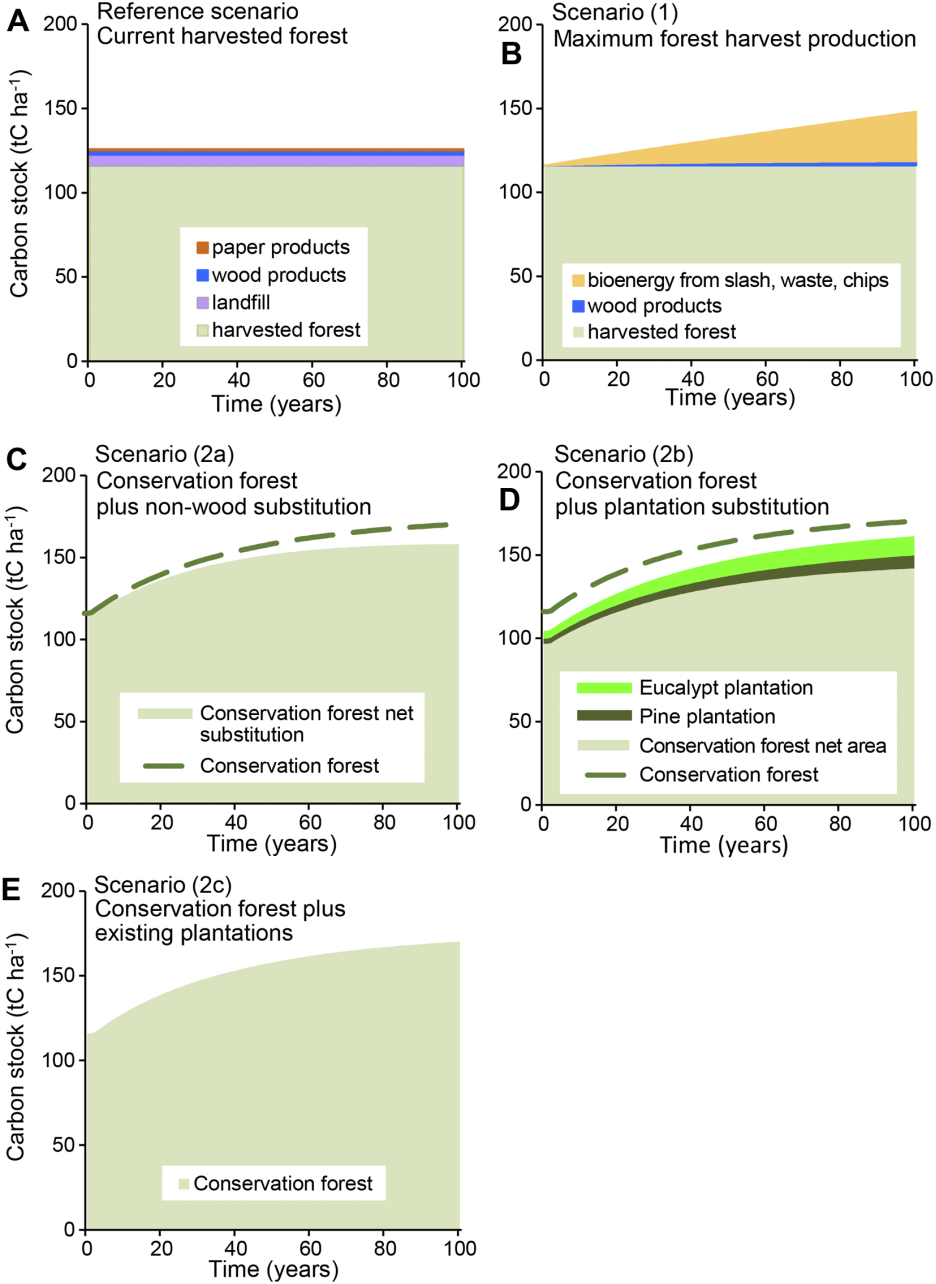
Regional average carbon stocks simulated over 100 years in South Coast mixed native eucalypt forest. Simulations were run for the reference case of current harvested forest (A), and four scenarios of forest management; scenario (1) maximum forest harvest production (B), scenario (2a) conservation forest plus non-wood substitution (C), scenario (2b) conservation forest plus plantation substitution (D), and scenario (2c) conservation forest plus existing plantations (E). All biomass pools in the harvested forest system were included, both on- and off-site. Carbon stock in harvested forest included above-and below-ground living and dead biomass. Carbon stocks shown for pine and eucalypt plantations included forest biomass living and dead, wood and paper products and landfill.

The simulation of changes in carbon stocks under current native forest management (reference scenario) was calculated at the local scale for the logged area on a rotation of 70 years (Fig A (A) in S3 Appendix), for the net harvested area on a return time of 20 years (a new selection of trees is harvested from the coupe after 20 years) (Fig A (B) in S3 Appendix), and at the regional scale as an average across the landscape (Fig 2). In the simulation of the average carbon stock across the region over a 100 year time period, the area under the curve represents the cumulative carbon stock in the forest system over time (Fig 2). Identifying the carbon stocks in each type of harvested area in this way provided an understanding of the dynamics over time and the mosaic of areas aggregated at the landscape scale. The simulated carbon stock change from our model was compared with modelled outputs from FCNSW [13] and productivity data reported for the species (Table A in S3 Appendix).

Scenarios where the native forest was harvested for wood products (Reference and Scenario 1) yielded a lower total carbon stock over 20, 50 and 100 year simulation periods than the scenarios where the native forest was conserved and wood products were substituted (Scenarios 2a, 2b, 2c) (Table 2). Biomass accumulation rate in the regrowing forest was calculated using two equations with different coefficients depending on the assumed maximum biomass set by the asymptote of the equation (Equations (S2-1) and (S2-2) in S2 Appendix S2.1.2). Carbon stocks calculated using Equation (S2-2), which was derived from the average of site data and has a higher maximum biomass value, predicted higher stocks in the conservation forest scenarios (Scenarios 2a, 2b, 2c).

**Table 2:** Total carbon stock (tC ha^-1^) in the harvested or conserved forest scenarios in South Coast NSW forests, simulated over 20, 50 and 100 years and calculated using two equations for biomass accumulation rate (S2 Appendix S2.1.2).

Forest management scenario		20 yrs	50 yrs	100 yrs
Biomass accumulation rate Equation (S2-1):				
Reference	Current harvested forest	126	126	126
Scenario (1)	Maximum forest harvest production	123	134	151
Scenario (2a)	Conservation forest plus non-wood substitution	137	152	158
Scenario (2b)	Conservation forest plus plantation substitution	128	148	162
Scenario (2c)	Conservation forest plus existing plantation area	139	158	170
Biomass accumulation rate Equation (S2-2):				
Reference	Current harvested forest	126	126	126
Scenario (1)	Maximum forest production	123	134	151
Scenario (2a)	Conservation forest plus non-wood substitution	137	161	186
Scenario (2b)	Conservation forest plus plantation substitution	128	155	186
Scenario (2c)	Conservation forest plus existing plantation area	139	167	198

### Mountain Ash native forest in the Central Highlands, Victoria

**Fig 3:**
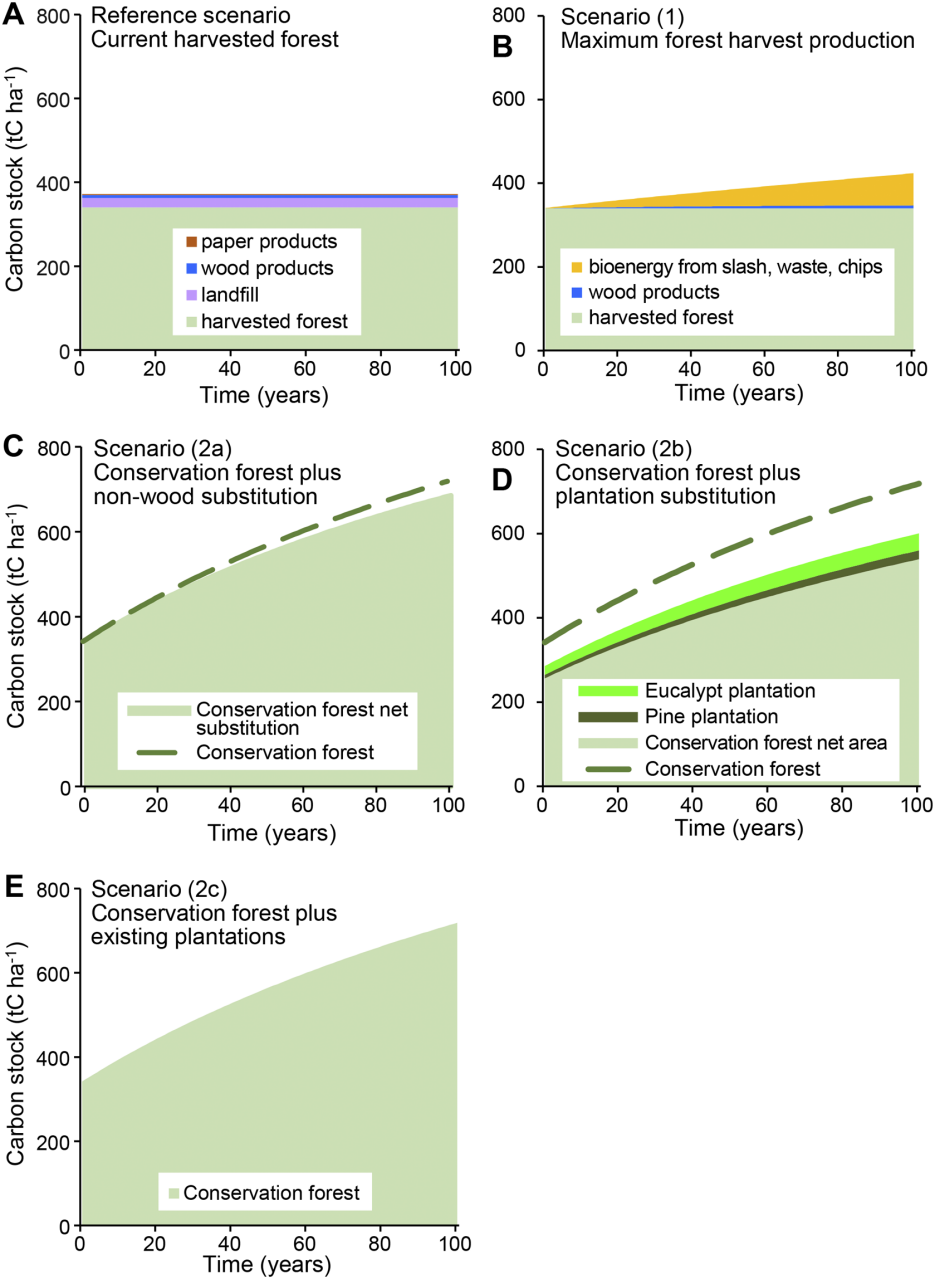
Regional average carbon stocks simulated over 100 years in Mountain Ash forest. Simulations were run for the reference case of current harvested forest (A), and four scenarios of forest management; scenario (1) maximum forest harvest production (B), scenario (2a) conservation forest plus non-wood substitution (C), scenario (2b) conservation forest plus plantation substitution (D), and scenario (2c) conservation forest plus existing plantations (E). All biomass pools in the harvested forest system were included, both on- and off-site. Carbon stock in harvested forest included above-and below-ground living and dead biomass. Carbon stocks shown for pine and eucalypt plantations included the forest biomass living and dead, wood and paper products and landfill. Forest carbon accumulation rate was calculated using Equation (S2-3) in S2 Appendix. See Fig D in S3 Appendix for calculation using Equation (S2-4) in S2 Appendix.

The simulation of changes in carbon stocks for the current native forest management of clearfelling (reference scenario) was calculated at the local scale for the logged area (Fig C in S3 Appendix), and at the regional scale as an average across the landscape (Fig 3)

Scenarios where the native forest was harvested for wood products (Reference and Scenario 1) yielded a lower total carbon stock over 20, 50 and 100 year simulations periods than the scenarios where the native forest was conserved and wood products were substituted (Scenarios 2a, 2b, 2c) (Fig 3 and Table 3). Biomass accumulation rate in the regrowing forest was calculated using two equations with different coefficients and assumed maximum biomass set by the asymptote of the equation (Equations (S2-3) and (S2-4) in S2 Appendix S2.2.2). Carbon stocks calculated using Equation (S2-3), which was derived from the average of site data and has a higher maximum biomass value, predicted higher stocks in the conservation forest scenarios (Scenarios 2a, 2b, 2c).

**Table 3:** Total carbon stock (tC ha^-1^) in the harvested or conserved forest system in Mountain Ash forests, simulated over 20, 50 and 100 years and calculated using two equations for biomass accumulation rate (S2 Appendix S2.2.2), and with a wildfire (Fig E in S3 Appendix).

Forest management scenario		20 yrs	50 yrs	100 yrs
Biomass accumulation rate Equation (S2-3):				
Reference	Current harvested forest	372	372	372
Scenario (1)	Maximum forest harvest production	359	385	424
Scenario (2a)	Conservation forest plus non-wood substitution	437	549	685
Scenario (2b)	Conservation forest plus plantation substitution	371	475	600
Scenario (2c)	Conservation forest plus existing plantation area	444	566	719
Biomass accumulation rate Equation (S2-4):				
Reference	Current harvested forest	283	283	283
Scenario (1)	Maximum forest harvest production	267	286	314
Scenario (2a)	Conservation forest plus non-wood substitution	325	406	499
Scenario (2b)	Conservation forest plus plantation substitution	264	336	418
Scenario (2c)	Conservation forest plus existing plantation area	330	418	523
Biomass accumulation rate Equation (S2-3), with wildfire:				
Reference	Current harvested forest	372	372	372
Scenario (1)	Maximum forest harvest production	359	385	424
Scenario (2a)	Conservation forest plus non-wood substitution	437	549	721
Scenario (2b)	Conservation forest plus plantation substitution	371	475	627
Scenario (2c)	Conservation forest plus existing plantation area	444	566	754

The effect of occurrence of a wildfire on the carbon stock dynamics over the 100 year simulation period was tested with a wildfire at year 56, which is half the estimated average return time for wildfires [52]. Emissions due to combustion were 10% of the biomass carbon stock and the carbon stock in the conservation forest after the fire consisted of regenerating vegetation, a small proportion of living trees, standing dead trees and coarse woody debris [50] (Fig E in S3 Appendix). Scenarios where the native forest was conserved, but burnt in a wildfire, and wood products were substituted still had the highest carbon stock, of living and dead biomass components, after the 100 year simulation period (Table 3).

### Sensitivity analysis

**Fig 4:**
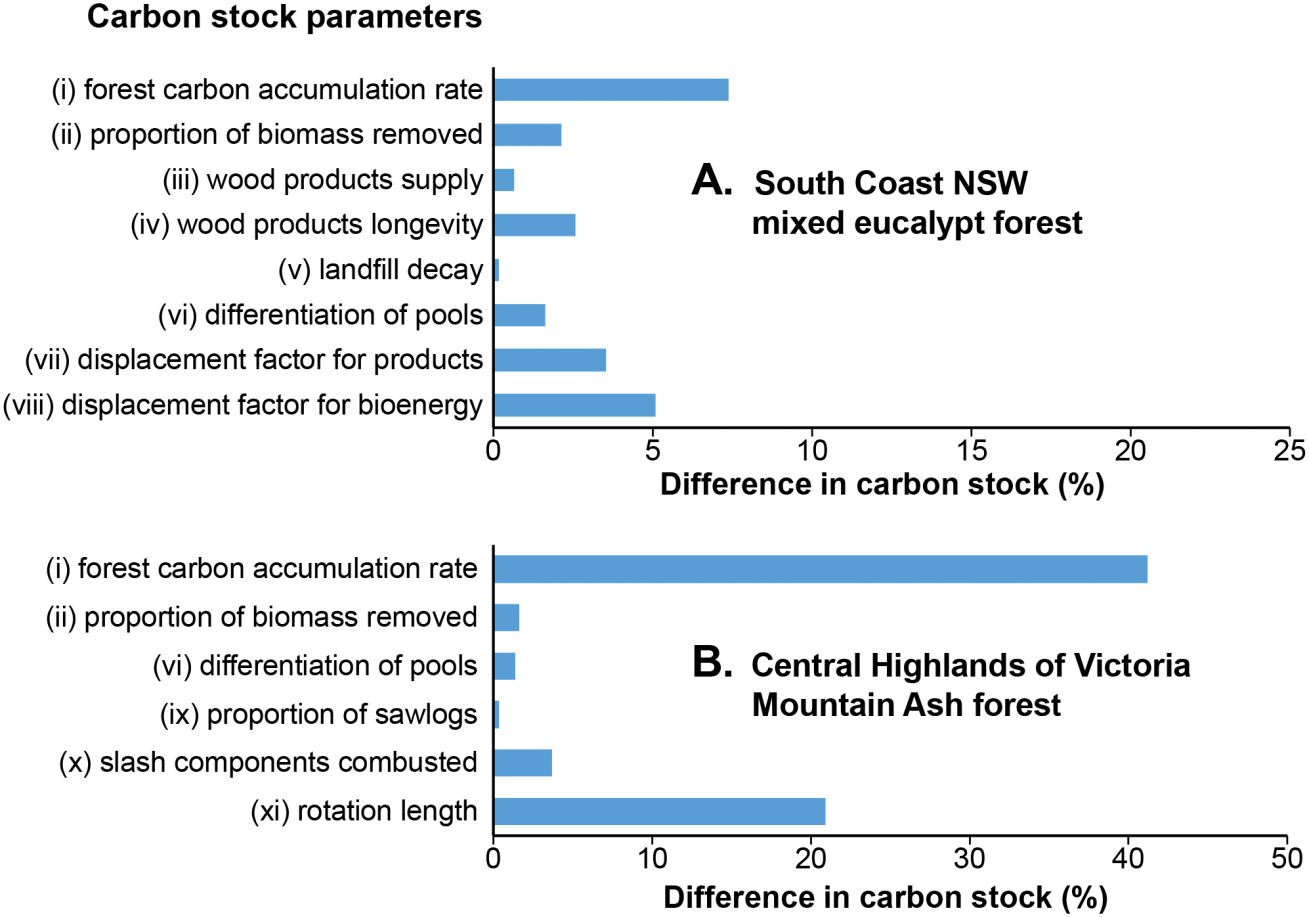
Differences in simulated carbon stocks (tC ha^-1^) as percentages of the total system carbon stock. Differences were calculated from use of minimum or maximum values of parameters listed in Tables B and C in S3 Appendix in a simulation for 50 years in (A) mixed native eucalypt forest on the South Coast of NSW, and (B) Mountain Ash forest in the Central Highlands of Victoria.

Differences in simulated carbon stocks due to each of the ten parameters tested in the sensitivity analysis are shown as percentage change in Fig 4 and as absolute values in Tables B and C in S3 Appendix. The simulated carbon stocks were most sensitive to parameters that determine the amount of biomass in the forest. In both case studies this parameter was the rate of carbon accumulation (parameter i), which resulted in a difference of 9 tC ha^-1^ after 50 years in the South Coast forest, and 148 tC ha^-1^ after 50 years in the Mountain Ash forest. Additionally, in the Mountain Ash forest, the rotation length (parameter xi) resulted in a difference of 75 tC ha^-1^ after 50 years.

The stocks of carbon in wood products and landfill remained small compared with that in forest biomass, irrespective of the wood volume produced, the types and longevities of wood products, and transfer to landfill. Parameters describing longevity of pools, and the proportion of biomass in long-lived pools, had the greatest influence on these carbon stocks. However, differences in rates of transfer of carbon through harvested products did not result in major changes in the total carbon stock (Fig 4iv, 4v, 4vi and 4ix).

The sum of the differences in carbon stocks due to the parameter values that determine the stocks in products, slash, landfill and substitution (Tables B and C in S3 Appendix), was lower for all simulation time periods than the difference in carbon stock resulting from the scenario of changing native forest management to conservation, for both forest case studies (Tables 4 and 5).

**Table 4:** Change in carbon stocks (tC ha^-1^) over the 20, 50 and 100 year simulation periods for scenarios of conservation forest with product substitution (Table H in S2 Appendix) compared with harvested forest plus products and landfill in NSW South coast forest. The difference in carbon stock due to scenarios is compared with the sum of the differences due to parameter values.

	Conservation forest			Harvested forest
	20 yrs	50 yrs	100 yrs	constant over time
Forest biomass	139	158	170	116
Products	-2.4	-6.0	-12.1	3.3
Landfill				6.5
Total	136.6	152.0	157.9	125.8
Difference due to scenarios (conservation—harvested)	10.8	26.2	32.1	
Difference due to sensitivity of parameter values	6.4	13.0	25.8	

**Table 5:** Change in carbon stocks (tC ha^-1^) over the 20, 50 and 100 year simulation periods for scenarios of conservation forest with product substitution (Table H in S2 Appendix) compared with harvested forest plus products and landfill in Mountain Ash forest. The difference in carbon stock due to scenarios is compared with the sum of the differences due to parameter values.

	Conservation forest			Harvested forest
	20 yrs	50 yrs	100 yrs	constant over time
Forest biomass	444	566	719	340
Products	-7.0	-16.9	-33.5	9.2
Landfill				22.5
Total	437	549	685	372
Difference due to scenarios (conservation—harvested)	65	177	313	
Difference due to sensitivity of parameter values	10.6	21.7	35.0	

None of the potential parameter values reported in the literature and tested in our analyses (Tables B and C in S3 Appendix) could increase the carbon stock in products, slash, landfill and substitution sufficiently to exceed the increase in carbon stock due to changing management of native forest to conservation.

## Discussion

### Relative mitigation benefit of native forest management strategies

We have demonstrated that changing native forest management from commercial harvesting to conservation can make an important contribution to climate change mitigation. Throughout the 100 year simulation period, the net carbon stocks were higher in the conservation scenarios (Scenario 2) than in the harvest scenarios (Reference case and Scenario 1), with the difference representing the net abatement from conservation. An important attribute of the abatement from avoided native forest harvesting is its upfront profile: stopping harvesting results in an immediate and substantial reduction in net emissions relative to the reference case where commercial harvesting continues. Because the economic returns from native forest harvesting are typically low or negative, and the abatement occurs upfront, the cost of mitigation from avoided harvesting is often less than other forms of land sector abatement, such as reforestation, where the climate benefits are incremental over long timeframes [53]. Mitigation benefits from forest management can be achieved by increasing the area and effectiveness of the protected area network and providing incentives for off-reserve conservation to maximise carbon stocks and biodiversity, and hence the resilience of ecosystems [4].

The climate change mitigation benefits derived from native forest conservation are mainly attributable to the fact that the largest carbon stocks and highest longevities of stocks occur in forest biomass (Tables 3 and 4). In the harvesting scenarios, only a small proportion of harvested wood products is transferred to pools with high longevities (Figs B and E in S2 Appendix).

While our results were based on two case study forest systems, the analyses tested a range of published parameter values. Hence, our conclusions are likely to be relevant to many other forest types. The results from our case studies are also in agreement with previous studies. Based on modelled simulations of different forest systems, Schlamadinger and Marland [54] concluded that conservation of forests and allowing regrowth provided the greatest carbon benefit for up to 100 years, even under different reference cases, growth rates and conversion efficiencies. After 100 years, the net carbon benefit was similar in many of the scenarios whether trees were used for harvest, energy and wood products, or whether they were conserved for their carbon stocks. Schlamadinger and Marland [55] also found there was a long-standing debt of net carbon emissions associated with a forest management system of harvested wood products and bioenergy due to the initial harvest of native forest with a large standing stock of biomass.

Additionally, the rules and procedures adopted by governments and international organisations for the accounting of greenhouse gas emissions, that is the policy institutions, influence the assessment of relative benefit of mitigation activities. Despite testing a range of institutional assumptions, the conclusion remained that greater mitigation benefit was gained by conserving forests in southern NSW than their sustainable harvest [56].

Our sensitivity analyses showed that parameters affecting carbon accumulation in forest biomass had a greater influence on total carbon stocks than those affecting transfers among wood products. This result has also been noted by Schlamadinger and Marland [9]. None of the parameters we tested that influence management of a harvested forest system could increase the carbon stocks to levels greater than the stocks in a conservation forest over simulations up to 100 years.

Native forests are most valuable for mitigation when conserved because their biomass carbon stocks continue accumulating under natural ecosystem processes of regeneration and growth [55,57]. Evidence for the higher carbon stocks in native forests than regrowth forests has been recognised in national policies [24]. Even in the early stages of negotiation of the Kyoto Protocol, Schulze et al. [58] proposed that the greatest net carbon benefit would be achieved by avoiding emissions from deforestation and forest degradation, and protecting existing native forests. To date, however, international forest-related policies, including negotiations under the U.N. Framework Convention on Climate Change, have failed to recognize fully the mitigation value of native forest conservation [59].

Use of harvested wood products from plantations, as commercial crops of trees, and regrowth forests [60] does provide storage of carbon and in theory can be substituted for more fossil fuel energy-intensive products. Wood products are a source of renewable construction materials that produce less greenhouse gas emissions per unit mass than most non-wood substitutes where fossil fuels are used in the production process or as the primary energy source, such as steel. Plantations combine storage of carbon in forest biomass with supply of products. Net carbon stock change assessed from a reference state of current land management as existing plantations or establishing plantations on previously cleared land, with plantations managed for production of wood and paper products, provides the optimal integration of storage of carbon combined with supply of products; noting that this does not account for the initial loss of carbon when the land was first cleared. The efficiency of plantation production is demonstrated by the total carbon dioxide emissions for logs from softwood plantations being 50–75% less than for hardwood logs from native regrowth forests in Australia [61]. Emissions per cubic metre of log harvested from Australian native hardwood forests were estimated to be among the highest in the world [61]. Carbon storage can be maximised in plantations by increasing the proportion of merchantable biomass, efficiency of processing, and longevity and recycling of products.

### Key issues influencing carbon accounting

During our analysis of changes in carbon stocks in harvested native forest systems, we identified key issues that influence the outcomes of carbon accounting. Incorporating these issues collectively in a conceptual accounting framework is beneficial for evaluating relative mitigation benefits of native forest management strategies. Mitigation benefits of forest management strategies should be assessed in terms of the long-term standing stock of carbon in the forest landscape and any changes over time in the net exchange of carbon with the atmosphere. Carbon stocks in biomass represent temporary storage that depends on the longevity and stability of the pools. These stocks will affect the concentration of carbon dioxide in the atmosphere only if there is a net change in the total biomass stock.

There is a lack of consistency in the treatment of some issues related to accounting of mitigation benefits. To improve understanding of the accounting system, clarity is required around the following six issues.

### Reference case

The reference case must be defined to provide the basis for comparison with alternative scenarios of forest management systems [54]. We analysed carbon stocks using the current regime of native forest management for commercial production as the reference scenario and compared this with two alternative scenarios of conservation native forests and harvested forests for maximum production of wood products and bioenergy. Defining explicitly the reference as the current condition of the forest is important for the comparison. For example, the biomass carbon stocks of a native forest subject to natural disturbance regimes but not logging will, by definition, be at their maximum or carrying capacity. By comparison, the biomass carbon stock in a regrowth forest subject to logging will be lower than the carrying capacity. At the landscape scale, stocks in regrowth forests may have a trend over time due to management activities, or may be at a steady state of growth and harvest removals.

Evaluating the impacts of native forest management activities must include quantification of carbon stocks in the natural ecosystem or primary forest [59] to determine the potential for sequestration by the regrowing forest to restore the carbon loss due to harvesting. Differences in estimation of sequestration potential have occurred because of assumptions about carbon accumulation rates in older forests. One view is that saturation of carbon stock occurs at forest maturity [13,62,63], whereas other estimates have shown continued accumulation of carbon in old-growth forests centuries old [58,64,65].

### Longevity of stocks

Carbon is transferred through plants, soil, products and waste materials. However, the average magnitude of these biomass pools and their longevity are the key parameters determining the contribution to mitigating atmospheric carbon dioxide concentration. Our sensitivity analyses demonstrated that changing the proportions of biomass utilised in different forms—slash, sawlog or pulp products, and landfill—had a relatively small influence on total carbon stocks (Fig 4). The longevities of these biomass pools were similar for various combinations of products. Combinations of sawlog (k = 0.0198 yr^-1^) and paper (k = 0.346 yr^-1^) products and processing waste (k = 1 yr^-1^) had similar longevities to that of slash (k = 0.0486 yr^-1^), and hence created little change in the total carbon stock (Tables D and E in S2 Appendix). Increasing the proportion of sawlog products and their input to landfill (k = 0.004 yr^-1^) did increase the carbon stock, but less than half of the biomass allocated to sawlogs was transferred to timber products because of the high proportion of processing waste (Figs B and E in S2 Appendix).

In contrast, the increased forest biomass from conserving native forest and allowing continued growth, increased the magnitude and longevity of the total carbon stock (Figs 2 and 3). This has also been shown in other forest types [7]. The carbon stocks of native forests are typically affected by natural disturbance events, such as fire, but these result in relatively small fluctuations due to emissions, with the carbon stock regained within a decade through regeneration, as shown in Fig E in S3 Appendix. Hence, the biomass carbon stocks in conserved native forests on a landscape basis can be considered as a stable stock with the value fluctuating in response to natural disturbances around a long term mean. Additionally, evidence from the 2009 wildfire in the Mountain Ash forest showed that protected old-growth forests were less likely to burn at high severity [66–68]. Even when the fluctuations in carbon stocks due to wildfires are included in long-term analyses of carbon dynamics, the conclusion remains that total carbon stocks are highest in conserved forests [7].

### Timeframes of accounting

The timing matters for activities to reduce emissions and contribute to climate change mitigation. The next decade is the critical time to implement mitigation activities if we are to limit global warming to less than 2°C above the pre-industrial global mean temperature; the internationally agreed target [24]. The benefit of reducing emissions depends on both the magnitude and timing of the occurrence of mitigation activities. A major benefit of changing native forest management activities to conservation as a form of mitigation is that implementation can be relatively rapid: there is an instant benefit from the avoided emissions that would otherwise have occurred; plus additional, albeit more slowly gained, benefits through sequestration from native forest restoration and reforestation [69].

The assumption of carbon neutrality of bioenergy, based on the equivalence of carbon fluxes in emissions and regeneration of the forest, is time-dependent. The time taken to replenish the initial biomass carbon stocks that were depleted by logging must be accounted for when evaluating mitigation benefit of forest management strategies. The time to repay this carbon debt will depend on the initial carbon stock and the rate of accumulation into woody biomass pools [25].

Our comparison of scenarios of carbon stock change in the NSW South Coast and Victorian Mountain Ash forests showed that over a 100 year simulation period, the highest stocks would be achieved from conserving native forests. Over longer time periods, and depending on the displacement factor, substitution of bioenergy may have a greater rate of increase in carbon stocks. However, given the urgency of the climate change problem [24], implementing forest management actions that increase emissions within, and only provide a benefit after, 100 years are not useful mitigation activities. Additionally, projections over many decades have a declining reliability, and displacement factors are likely to decrease over time.

### Storage in landfill

The carbon storage capacity of landfill depends on the rates of decay of waste material, which is one of the most difficult parameters to estimate. We used the default value from Australia’s National Carbon Accounting System (NCAS) [35], which was derived from measurements of decayed material in landfills that were only up to 46 years old, or just over one half-life for wood [36]. However, the IPCC [70] recommends that data on rates of decay should be collected over time period equivalent to 3 to 5 half-lives to achieve an acceptable accuracy of the results. The difficulty in estimating this parameter and consequent wide range in values contributes to uncertainty in carbon accounts (Table E in S2 Appendix).

The scenario of maximising carbon storage in harvested forest systems relies on the long-term storage of waste material in landfill. Only a proportion of wood and paper products are transferred to landfill (0.44 to 0.95 depending on product type [35]), and of this amount, proportions of 0.23 for wood and 0.49 for paper products decompose (Table E in S2 Appendix). The proportion of the initial forest carbon stock that remains in long-term storage in landfill is less than 3% (Figs B and E in S2 Appendix).

Additionally, other issues with landfills may make long-term storage not a sustainable option. Landfills occupy large areas of land, often near urban areas, so that disposal of waste in ever-increasing areas of landfills has become non-viable [71]. Many regional and national policies aim to minimise waste disposal [72]. In Australia, the proportion of wood waste transferred to landfill is decreasing and the proportion utilized for combustion is increasing [18]. Both carbon dioxide and methane are produced during decomposition, and about a third of the methane generated is captured or oxidised before release [35]. Additionally, the stability of carbon stocks in landfill is uncertain. The very low rates of decomposition reported are based on anaerobic conditions, but these conditions cannot be assured in the future. Decomposition would increase if the site became aerobic. However, there is an increasing trend for methane capture and combustion. The potential for changed conditions for decomposition in the future creates a great risk for greenhouse gas emissions [8]. Use of wood product waste for bioenergy instead of input to landfills would provide a helpful substitution for fossil fuel energy sources [73].

### Substitution of products and energy

Wood products are a renewable source of construction material and energy. Hence, their utilisation potentially can provide mitigation benefits if they substitute for more emissions-intensive products. However, increasing sequestration in the land sector requires use of wood products and/or bioenergy that are additional to the current forest harvesting regime [74–76]. This means an increase in productivity, by increasing the area of forest harvested, the intensity of harvesting, or the efficiency of product utilisation. The capacity for additional production in terms of forest area available and wood yield would need to be demonstrated, and competition with other land uses evaluated. A simulation of potential carbon sequestration in wood products and landfill should start at the current level of production, not zero, and predict the potential for additional production and carbon storage. Foregone fossil fuel-based products and energy sources must be demonstrated.

We used constant displacement factors for wood products and bioenergy in our simulation based on current rates. However, it is likely that these factors will decrease over time as limits exist to the amount of substitution of wood products due to the effects of market and policy factors. Additionally, abatement opportunities are being sought in all sectors of the economy to meet mitigation obligations, which are likely to increase use of other renewable energy sources and increase energy efficiency, thereby driving down displacement factors over time.

Biomass is a renewable form of energy but it is not carbon neutral. An opportunity cost is incurred from the loss of the initial carbon stock and potential for carbon sequestration when a forest is used for harvested products [75,76]. Additionally, loss of carbon occurs through processing waste, and energy is required for processing and transport. Biomass has a higher rate of carbon emissions per unit energy produced than oil or natural gas, and biomass is usually burned with a lower efficiency than fossil fuels [77–80]. In fact, quantification of the global warming potential of carbon dioxide emissions from combustion showed that bioenergy derived from slow-growing forests was higher than that from fossil fuels over the whole period for at least a 100-year time frame [81]. Additionally, provision of wood for bioenergy from existing harvested forest systems means increasing the intensity of harvesting or utilising the slash. Removing additional residue material, particularly foliage, bark and small branches, increases nutrient removals and likely impacts soil organic matter, so that the sustainability of the practice would need to be carefully monitored [78,82].

The value of substituting wood products is only valid if the alternative products are more fossil fuel intensive to produce. Both the use of fossil fuel in the production process, such as coal in the case of steel, and as the energy source in their manufacture, must be considered. If non-fossil fuel sources are used as raw materials and energy for manufacturing then there would be no mitigation benefit from using wood products. Wood products have lower embodied energy than many other products but their substitution provides only a temporary store of carbon [77].

### Boundaries of the system

In our analysis, we have confined the boundaries of the system to the physical transfer of carbon and ensured comprehensiveness in accounting for the stocks. However, analysis of the broader system required for land use decision-making should include socio-economic, institutional and land use management factors that influence the transfers of carbon stocks.

In the current study, as in many other studies (for example [9,12,13]), direct substitution of products and energy for fossil fuel emissions was assumed. This means that the cessation of native forest harvesting necessarily results in substitution by non-native forest wood products and energy sources. However, consideration should be given to the supply and demand for products, potential sources of alternative products, markets and regulatory factors to assess feasibility of the substitution.

Substitution by bioenergy must directly displace fossil fuel emissions to be of benefit for mitigation [26]. Direct substitution may not occur where demand for energy exceeds current supply in developing countries because sources of bioenergy are likely to increase energy consumption, not reduce fossil fuel emissions [55]. Where there are national policies that guarantee a specified amount of renewable energy (for example, a ‘renewable portfolio standard’), increases in bioenergy are likely to replace other forms of renewable energy such as solar and wind, and so not reduce emissions [56]. Global empirical analysis of the use of bioenergy has shown displacement of fossil fuel sources is much less than unity [83]. Additionally, practicalities of the market must be considered, such as transport distance from product source to energy generator.

The land resources required to produce wood products and the opportunity costs of alternative land uses, such as production of food and fibre and human settlements, need to be considered within regional land use management decisions [77]. Very large land areas would be required to make a significant contribution to climate change mitigation through bioenergy substitution, and this would compete with other land uses and put pressure on existing forests. An assessment of the land resources required globally to produce biomass for biofuel substitution of transport fuels concluded that areas of land were not available on the scale required [11]. Finally, we note that photovoltaic cells are more efficient than the photosynthetic processes in plants at converting sunlight to forms of energy that can be used (about 1% compared to 6–21% [82,83]). Not only does bioenergy therefore require large areas of land, it also needs fertile and well-watered land; the same land needed for growing food.

## Conclusions

The mitigation benefit of carbon stock changes due to forest management is determined by the net effect on the atmospheric carbon dioxide concentration over time [77]. Based on this assessment, we found that the greatest mitigation benefit from native forest management, over the critical decades within the next 50 years, is achieved by protecting existing native forests. None of the potential parameter values, which create uncertainty in quantifying transfers of carbon stocks, could increase the stock in products, slash, landfill and substitution sufficiently to exceed the carbon stock in the conservation native forest. While data to define some carbon stocks and stock changes are limited, we attempted to apply the greatest range of potential parameter values available from current data sources and the literature, in order to test the results and identify the consequences of different assumptions.

Wood products are valuable stores of carbon compared with the sources of emissions from other construction materials; but most wood products can be sourced from plantations. Currently, 84% of log volume is harvested from plantations in Australia [29]. Substitution of fossil fuel energy is best achieved by using non-carbon renewable energy sources like solar, wind, tidal and geothermal power, which also require less land area. Wood has many benefits as a construction material; but it does not have a climate change mitigation benefit compared with conservation of native forests.

## Supporting Information

S1 Appendix References about mitigation benefits of forest management in the literature. (PDF)

S2 Appendix Input data for calculation of forest carbon stock. (PDF)

S3 Appendix Results. (PDF)

S4 Appendix References for Supporting Information. (PDF)
