# Supplementary material for: Under What Circumstances Do Wood Products from Native Forests Benefit Climate Change Mitigation?
Source: PLoS One. 2015 Oct 5;10(10):e0139640. doi: 10.1371/journal.pone.0139640 (PMC4593608; doi:10.1371/journal.pone.0139640)
Supplement: S1 Appendix — (PDF) [file pone.0139640.s001.pdf]

S1 Appendix. References about mitigation benefits of forest management in the literature

SI Table A. Summary of reports in the literature about relative mitigation benefits of different forest management systems.
Time for zero net benefit (ZNB) is the time for the carbon stock in the harvested forest area plus associated wood and bioenergy products to regain the level of the pre-harvest carbon stock.

Forest system	Products	Time for zero net benefit	Reference
Harvested native forest: old-growth and regrowth	Wood products	>200 yrs	Harmon et al. (1990)
Harvested forest (growth rates < 1.2 tC ha^{-1} yr^{-1}, initial biomass > 275 tC ha^{-1})	Wood and bioenergy, energy conversion efficiency < 60%	>100 yrs	Sclamadinger and Marland (1996a)
Regrowth forest	Maximum product and bioenergy substitution	approx. 100 yrs	Sclamadinger and Marland (1996a)
Harvested native forest	Wood products and bioenergy	55 yrs	Ximenes et al. (2012b)
Harvested forest	Wood products and bioenergy	30-40 yrs or 100 yrs depending on system	Marland and Schlamadinger (1995)
Harvested forest	Wood products and bioenergy	100 yrs	Marland et al. (1997a)
Harvested forest	Wood product substitution	90 yrs	Lippke et al. (2011)
Reforestation short rotation	Energy crop	150 yrs	Marland and Schlamadinger (1997)
Harvested forest	Bioenergy, substitution efficiency >50%	100 yrs	Kirschbaum (2003)
Harvested forest	Bioenergy substitution	>100 yrs	Schlamadinger and Marland (1996b)<br>Marland and Schlamadinger (1995)
Harvested forest	Wood products and bioenergy	40 yrs for regrowth, 100 yrs for protected forest	Marland and Sclamadinger (1995)
Short rotation	Biofuel	after 30 yrs, 2 to 9 times less than ZNB	Righelato and Spracklen (2007)
Harvested forest	Wood product substitution	90 yrs	Perez-Garcia et al. (2005)
Old-growth forest harvested then regrowth rotations	Wood products	400 – 2000 yrs	Dean et al. (2012a)
