# Supplementary material for: Under What Circumstances Do Wood Products from Native Forests Benefit Climate Change Mitigation?
Source: PLoS One. 2015 Oct 5;10(10):e0139640. doi: 10.1371/journal.pone.0139640 (PMC4593608; doi:10.1371/journal.pone.0139640)
Supplement: S2 Appendix — (PDF) [file pone.0139640.s002.pdf]

S2 Appendix. Input data for calculation of forest carbon stock

S2.1 Mixed eucalypt forest on the South Coast of NSW

S2.1.1 Biomass carbon stock in the forest

Current biomass carbon stock in the forests across the South Coast region was estimated from Forestry Corporation of NSW's (FCNSW) inventory data, which account for the age distribution of stands resulting from varying times since harvesting and disturbance events. The inventory data provided tree volumes for each forest type (Ximenes et al. 2012a), and these data were converted to aboveground living biomass carbon stock using species-specific basic density of wood for each forest type (Illic et al. 2000) and carbon concentration (Gifford 2000). An aboveground biomass carbon stock of 80.4 tC ha^{-1} was calculated as the area-weighted average of the forest types and age distribution within the region. This average stock was assumed to remain stable during our simulation period under a sustainable harvesting regime where log volumes removed are replaced by regrowing biomass across the mosaic of forest age class distribution within the region. Belowground biomass was estimated from a default root : shoot ratio of 0.2 for eucalypt forests (Snowdon et al. 2000), and dead biomass was estimated from the default value of 20 tC ha^{-1} used in Australia's National Carbon Accounting System (NCAS) for this forest type (Woldendorp et al. 2002, NIR 2012). From a total biomass of 116 tC ha^{-1}, the aboveground living biomass was assumed to vary over time due to harvesting but the dead biomass and belowground biomass remained constant (Table A).

S2 Table A: Current carbon stock in forest biomass averaged for the forest types in the South Coast region.

Parameter	Initial Data	Data	Reference	Calculation	Value
Current carbon stock per ha	1.a) Live standing volume from FCNSW inventory data for seven forest types<br>b) Species specific wood density	230.9 m^3 ha^{-1}<br>0.700 t m^{-3}	Ximenes et al. (2012a)<br>Illic et al. (2000)	Area-weighted average carbon stock for the seven forest types occurring in the region.	Aboveground biomass 80.4 tC ha^{-1}
	2. Average from simulation graph over 200 years		Ximenes et al. (2012a)		Aboveground biomass 82.8 tC ha^{-1}
	3. Root : shoot ratio	0.2	Snowdon et al. (2000)	Living aboveground and belowground biomass	Total living biomass 96 tC ha^{-1}
	4. Estimates of dead biomass for forest types	20 tC ha^{-1}	Woldendorp et al. (2002)	Total living plus dead biomass	Total biomass 116 tC ha^{-1}
Current carbon stock in the region	a) Net harvestable area<br>b) Aboveground biomass carbon stock density	96,513 ha<br>80.4 tC ha^{-1}	FCNSW (2009)	Area multiplied by average carbon density	Total carbon stock 7,759,645 tC

S2.1.2 Biomass accumulation rate in the forest

Accumulation of biomass carbon stock over time in regrowing forest stands after harvesting was described using equations derived using the Chapman-Richards form of the growth equation (Richards 1959, Janisch and Harmon 2002). The equation was fitted to available inventory data of biomass in South Coast forests of different ages up to 80 years (Fig A open diamonds) (Furrer 1971 cited in Borough 1984). The biomass yield estimated by Furrer (1971) concords with the relationship between tree size and age for *Corymbia maculata* up to 140 years old (Ash and Helman 1990). Two functions were derived using different assumptions for the asymptote that represents maximum carbon stock; 130 tC ha^{-1} (solid black line) as stated by Ximenes (2012b), and 250 tC ha^{-1} as a conservative estimate from site measurements in undisturbed forest (Table B) (dashed black line) (Fig A). Additional site data of biomass carbon stocks (Table B) are shown on the graph to illustrate the range in values for (i) undisturbed sites (black squares), whose average is approximately 250 tC ha^{-1} (assigned a nominal age of 200 years to approximate an old-growth forest), and (ii) sites that had been selectively harvested at an unknown time (black circles) (assigned a nominal age of 100 years indicative of a little greater than the age of harvest maturity at 70 years). Accumulation of biomass was described by the following equations:

$$\text{AGB (tC ha}^{-1}) = 130 * (1 - \exp(-0.022 * age))^{\wedge 0.52} \qquad \text{(S2-1)}$$

$$\text{AGB (tC ha}^{-1}) = 250 * (1 - \exp(-0.003 * age))^{\wedge 0.45} \qquad \text{(S2-2)}$$

where, AGB is aboveground living biomass, and *age* is the predominant age of the trees at the site. This biomass accumulation rate is compared with the simulated carbon stock in a ‘conservation forest’ used by Ximenes et al. (2012b) (dotted black curve), which does not reach the stated maximum after 200 years. Equation (S2-1) was used in the base case simulations for the South Coast forest to provide a conservative estimate of biomass. However, we consider that the higher biomass predicted in older forests by Equation (S2-2) is closer to the values of the field data, and this was tested in the sensitivity analysis (Table B in S3 Appendix).

S2 Table B: Biomass carbon stocks from field sites in the South Coast region. A aboveground biomass, T total biomass

Parameter	Location	Stand living biomass (tC ha^{-1})	CWD (tC ha^{-1})	Reference
1. Native forest (maximum modelled)	South Coast	130 (A)		Ximenes et al. (2012a)
2.Native forest (maximum measured)	South Coast	110, 143, 198 (A)		Ximenes et al. (2004)
3.Native forest	Kioloa (n=285 points)	250 (A), 294 (T)		Ash and Helman (1990)
4.Native forest (undisturbed)	Batemans Bay (n=12 sites)	447 (A), 538 (T)	53	Woldendorp (2000)
5.Native forest (undisturbed)	South Coast (n=4 sites)	270 (A), 328 (T)	58	Mackey (pers. comm.)
6.Native forest (some logged sites)	Kioloa (n=44 sites)	400 (A), 480 (T)	38	Jones (1998)
7.Native forest (average of harvested sites)	Kioloa (n=17 sites)	211 (A)	63	Roxburgh et al. (2006)
8.Native forest (estimated maximum)	Kioloa	364 (A)	81	Roxburgh et al. (2006)
9.Native forest (probably some harvesting)	Bega (n=5 sites)	165 (A)		Turner et al. (1992)
10.Regrowth mature native forest	Eden (n=3 sites)	218(A)		Turner and Lambert (1986)
11.Regrowth native forest after selective harvesting	Batemans Bay (n=1 site)	143 (A)		Ximenes et al. (2004)
12.Regrowth native forest after selective harvesting	South Coast (n=1 site)	107 (A)		Florence (1996)
13.Native eucalypt forest (no disturbance)	Spatial average for SE Australia	241 (A), 289 (T)		Keith et al. (2010)

S2 Fig A. Accumulation of aboveground biomass carbon in regrowing South Coast forest.

Equation (S2-1) was derived from the maximum carbon stock as stated by Ximenes et al. (2012b) (solid black line). Equation (S2-2) was derived the maximum carbon stock based on site measurements in undisturbed forest (dashed black line). The simulated carbon stock in a 'conservation forest' was used by Ximenes et al. (2012b) (dotted black curve).
Site data of biomass carbon stocks included: undisturbed sites (black squares), sites selectively harvested at an unknown time (black circles), and inventory data of biomass up to 80 years (open diamonds).

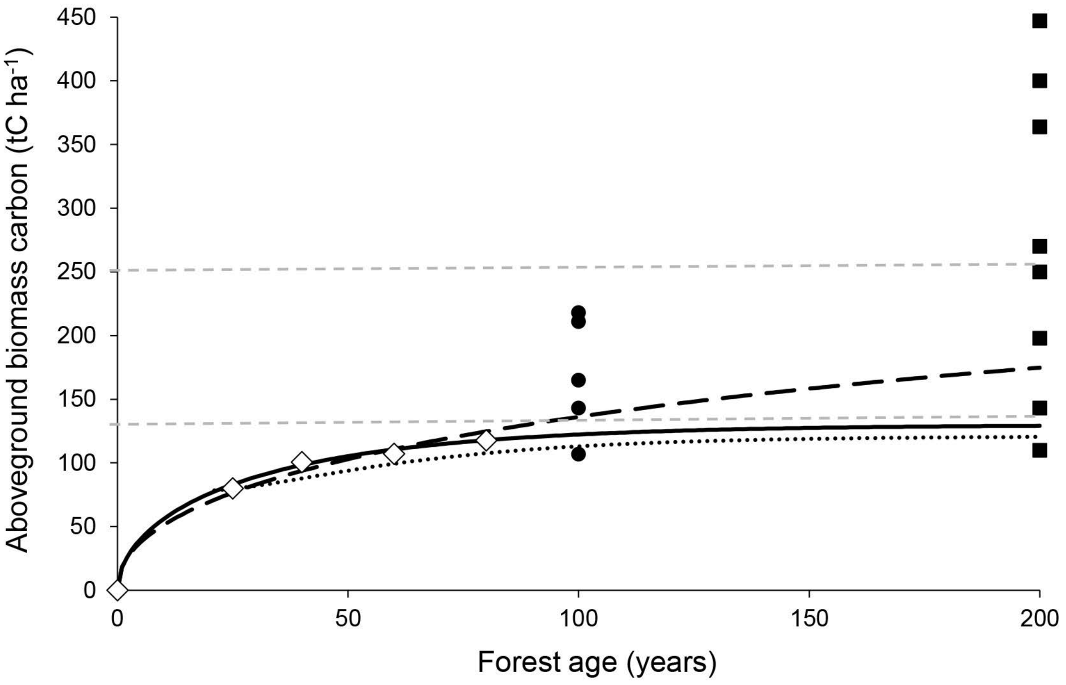

S2.1.3 Simulation of carbon stock change in harvested forests

Due to the silvicultural system of partial harvesting, changes in carbon stock per unit area are difficult to estimate. Hence, we analysed changes in carbon stocks in three ways.

(i) *Logged area.* The logged area is a patch within a coupe (cutblock) selected for logging. All trees are harvested and it is assumed that the aboveground living biomass is then zero. Harvesting return time to individual patches was 70 years (IFOA 2013) and the aboveground biomass at harvest maturity was 115 tC ha^{-1}.

(ii) *Net harvestable area.* The harvesting regime within the net harvestable area removes an average of 0.275 of the biomass in each harvesting event on a rotation of 20 years (Florence 2007), that is, 5% of the area is harvested each year (Table C).

(iii) *Regional average.* The silvicultural system was assumed to represent sustainable harvesting, such that the total biomass carbon stock in the standing forest remained stable

across the age class distribution within the region. Carbon stocks were calculated as tonnes of carbon per average hectare of forest in the net harvestable area of the region. An average hectare represents forest of an average age class distribution and frequency of harvesting.
The areas logged in the South Coast are quantified in these three ways (Table C).

S2 Table C: Areas logged in the State Forest in the South Coast sub-region of Forestry Corporation of NSW.

Description of area	Area (ha)	Reference
Gross area	199,093	FCNSW(2009)
Net harvestable area (NHA)	96,513	FCNSW (2009)
Actual net harvestable area per year averaged for 2005 - 2013	4778<br>~5% of total NHA	IFOA (2013)
Actual logged area per year averaged for 2005 - 2013	2949	IFOA (2013)

S2.1.4 Harvested wood products

Biomass removed off-site as wood products was assessed as the yield of carbon stock per net harvestable area or coupe (cutblock). Yield was estimated using data from different sources. Forestry Corporation of NSW harvest plans for each coupe in the region detail the expected product volume yield in the coupe area and we calculated the average yield for the current harvest plans. The NCAS used existing expert knowledge of the silvicultural systems of the region and FCNSW data to estimate average yields that had been achieved in harvesting operations historically (Florence 2007). We calculated area-weighted average values for the forest types in the South Coast sub-region reported in Ximenes et al. (2012a). Input data used to calculate yield are given in Table D.

The proportions of biomass carbon stock transferred in each stage of the harvested wood products system were derived from FCNSW data (Fig B). At the first stage of harvested wood products removed off-site and slash remaining on-site, the proportions differ depending on initial conditions. Initial conditions used for the calculation include: (i) log mass in commercial trees removed off-site compared with the aboveground living biomass of all trees on-site is 0.22 (calculated from Ximenes et al. 2004); (ii) log mass in commercial trees removed off-site compared with the aboveground living biomass of commercial trees on-site is 0.61 (calculated from Ximenes et al. 2004); and (iii) the default value for a medium-dense eucalypt forest used in the NCAS is 0.35 of aboveground biomass is removed off-site (NIR 2012). We used the NCAS value in the base case of the simulations and then tested the effect of these different proportions on the resulting changes in carbon stocks. The biomass that is not removed as product remains on-site as slash or waste material. This slash decomposes at a rate of 0.0486 yr^{-1}, based on estimates from coarse woody debris decomposition (Mackensen and Bauhus 1999).

The proportions of sawlog and pulp products, their processing and longevity are derived from FCNSW (2011) and Ximenes et al (2012a) yield forecasts over the next 100 years. Wood products in service includes 0.15 that is recycled at the end-of-life of green structural and dried and dressed sawn timber.

S2 Table D: Input data for the calculation of yield of biomass removed off-site for wood and paper products.

Estimated wood product removed	Value	Derivation	Reference
**Total for sub-region**	(tC yr^{-1})		
Actual harvest	56,190	Recorded harvest volumes average from 2005 to 2013	IFOA (2013)
**Carbon stock yield**	(tC ha^{-1})		
Future harvest	12.4	Planned harvesting schedule, projected log production per net harvest area	FCNSW (2013) Compartment Harvest Plans
Average past harvesting	14.6	Estimated past harvesting rate, area weighted average by forest type	Florence (2007)
Actual harvest	13.0	Recorded harvest volumes per year averaged from 2005 to 2013	IFOA (2013)
**Proportion of biomass harvested**			
Proportion of tree basal area harvested	0.277	Estimated past harvesting rate, area weighted average by forest type	Florence (2007)
Proportion of biomass harvested	0.275	Biomass estimated in product plus slash (22.1 tC ha^{-1}) divided by average aboveground biomass (80.4 tC ha^{-1})	Florence (2007) Ximenes et al. (2012a)
Proportion of biomass harvested	0.23	Estimated from average pre-logging standing volume and volume removed by selective harvesting	Florence (1996)
Maximum proportion of biomass harvested	0.40	Defined as a maximum basal area removal	Ximenes et al. (2012a)
Return time for harvesting within a net harvestable area	20 years		Florence (2007)
Return time for harvesting individual trees	70 years		IFOA (2013)
**Proportion of harvested biomass removed as product**			
Proportion from a commercial tree	0.607	Proportion of product from a selected commercial tree	Ximenes et al. (2008a)

		harvested (mean for *E. fastigata* and *C. maculata*)	
Proportion from a commercial tree	0.45–0.65	Proportion of product from a selected commercial tree harvested, range from five species	Ximenes et al. (2008a)
Proportion from the aboveground biomass of a forest	0.22	Range of values from 17% to 32% (for different stand qualities) calculated from data in Appendix 5 and Table 4 for all trees in a logged area	Ximenes et al. (2004)
Proportion from the aboveground biomass of a forest	0.35<br>0.22–0.53	Mixed coastal forest NSW<br>Range of values used in the FullCAM forest type plots	NIR (2012)
Proportion from the aboveground biomass of a forest	0.5<br>0.3–0.75	Average for harvested native forests in Australia	Farine et al. (2012)
Proportion from the aboveground biomass of a forest	0.5–0.7	Average for US harvested forests	Lippke et al. (2011)
**Proportion of sawlogs going into products**			
Native hardwood logs	0.36	Average for native forest regions in Australia	Tucker et al. (2009)
**Proportion in different wood products**			
Paper, cardboard, packaging<br>Structural timber<br>Panels, chipboard	0.5<br><br>0.2<br>0.3	Average values for European wood products	Reid (2004)
**Longevity of wood products** (years)			
Longevity of product	6 – 100	Full range of wood products	Skog and Nicholson (2000)
Lifespan or maximum age for the product pool and the time for decay of DOC_f.	30<br>50<br>90	$k = 0.10\ yr^{-1}$<br>$k = 0.06\ yr^{-1}$<br>$k = 0.033\ yr^{-1}$	NIR (2012)
Half-life of average wood products	$t_{0.5} = 30$	$k = 0.023\ yr^{-1}$	IPCC (2006)
Half-life of:<br>sawn timber<br>wood panels	 $t_{0.5} = 35$<br>$t_{0.5} = 25$	 $k = 0.0198\ yr^{-1}$<br>$k = 0.0277\ yr^{-1}$	UNFCCC (2012)
Average lifetime:<br>Structural timber<br>Average timber products	 75<br>33		European Wood Factsheet (2014)
Average lifetime of structural timber in houses	80		Lippke et al. (2011)<br>Perez-Garcia et al. (2005)

S2 Fig B. Transfers of carbon stocks in a harvested forest on the South Coast of NSW.

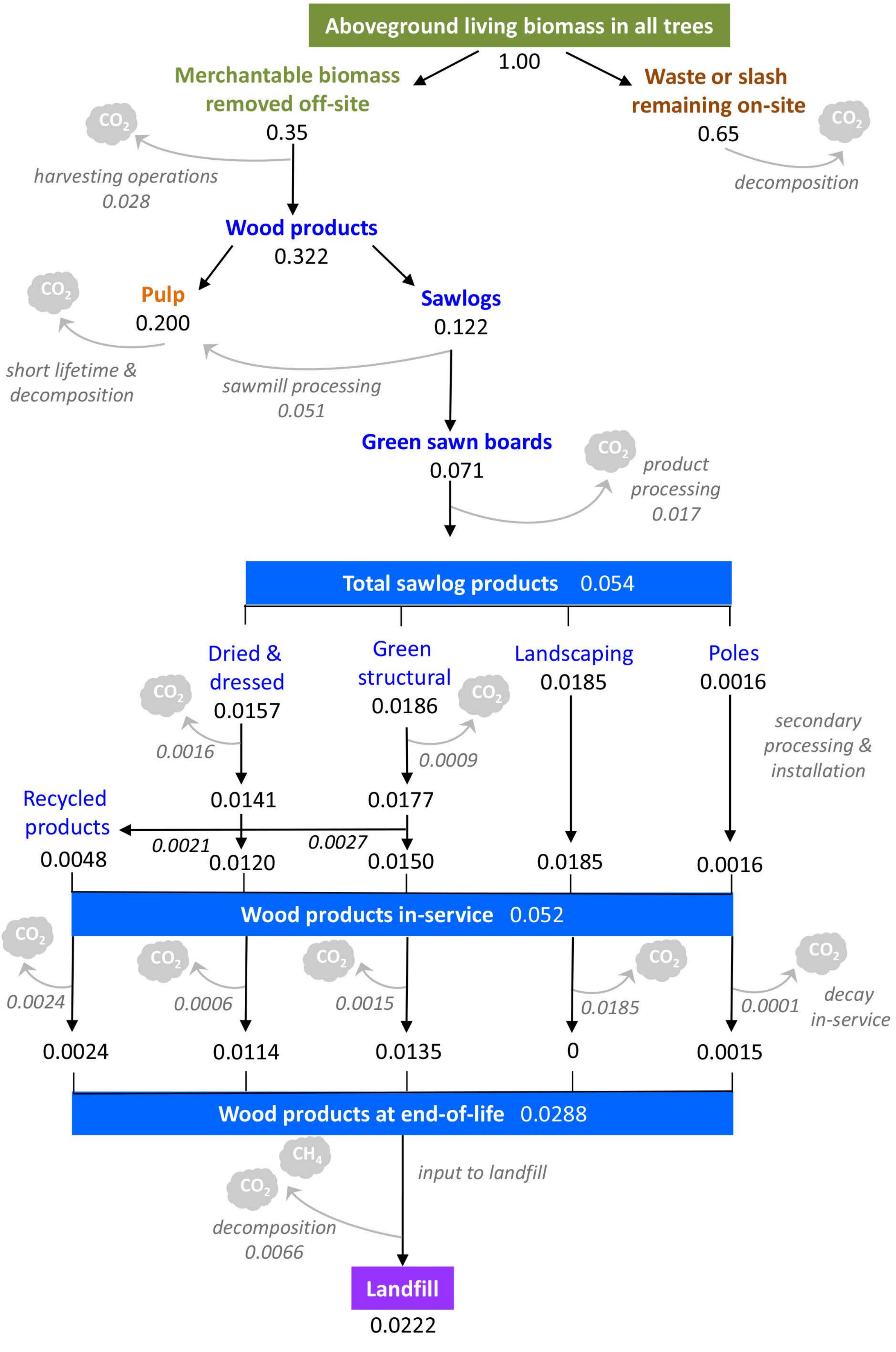

S2.1.5 Landfill

At the end-of-life of wood and paper products, the carbon stock becomes a waste material that is either recycled, combusted or stored in landfill. The magnitude of the stock in landfill depends on the rate of input of waste material and the rate of its decomposition. The values for these input data are derived from experimental data and default values used in accounting systems (Table E).

Input of material to landfill was determined from the longevity of wood and paper products, and the proportion of these products at end-of-life transferred to landfill. Conditions in a covered landfill are considered to be anaerobic and so decomposition occurs by bacterial rather than fungal activity. Bacteria can break down free cellulose and hemicellulose but not lignin or materials protected within lignin structures. Lignin comprises a quarter to a third of the carbon content of eucalypt wood (Wang et al. 2011). Decomposition under these reduced conditions results in emissions of approximately equal proportions of carbon dioxide and methane. We have accounted for emissions from landfill as a change in carbon stock, but have not taken into account the 25 times greater global warming potential of methane per unit carbon over a 100 year time horizon (equivalent to 72 times greater over a 20 year time horizon) (Forster et al. 2007).

Three forms of data were used to model emissions from decomposition in landfill: (i) the maximum degradable organic carbon tested under optimal experimental conditions (Barlaz 1998, 2004), but the timeframe for decomposition was not determined; (ii) the rate of decay of the degradable fraction (IPCC 2006), but any fraction that never decomposes was not determined; and (iii) the proportion decayed over a given time (Ximenes et al. 2008b), but this did not indicate change in rate over time.

The fraction of material that is potentially decomposable (DOC_f) in the long term was determined from tests under anaerobic conditions in laboratory reactors operated to obtain maximum methane yields. This represented an upper limit of the decay process (NIR 2012). Values estimated for DOC_f included 0.081 to 0.157 for a range of wood types (Wang et al. 2011), 0.18 for hardwoods (Ximenes et al. 2008b), and 0.23 for branchwood (Barlaz 1998). Various rates of decay have been estimated. Experimental decay of eucalypt wood under optimum conditions in the laboratory resulted in no production of methane because of toxic leachates (Wang et al. 2011). Estimates of decay rates derived from excavations of landfills in NSW, which had been closed for certain time periods up to 46 years, yielded an average of 0.004 yr^{-1} (Ximenes et al. 2008b). Estimates from experimental studies of decay rates internationally range from 0.02 to 0.07 yr^{-1} (Table E). These estimated decay rates result in a range of turnover times ($t_{0.95}$) for landfill from 152 years (IPCC 2006) to 350 years (Ximenes et al. 2008b). We used the values of DOC_f = 0.23 and k = 0.004 yr^{-1} for wood products and DOC_f = 0.49 and k = 0.05 yr^{-1} for paper products in the base case simulation and then tested the sensitivity of carbon stock change with a range of these rates of transfer in landfill. Stocks in landfill and rates of decomposition were treated in the same way in the simulations for each of the case study forest types.

S2 Table E: Input data for calculation of carbon stocks in landfill, including rates of waste material input and decomposition.

Values in bold were used in the current analysis.

Parameter	Derivation	Value	Reference
Proportion of wood products that goes to landfill	NSW Department of Environment and Conservation data	0.7	Ximenes et al. (2006)
	Municipal solid waste going to landfill in the US	0.7	Barlaz (2006)
	End-of-life wood products	0.67	Lippke et al. (2011)
	Data for destination of wood products at end-of-life	**0.85**	NIR (2012)
Fraction of degradable organic carbon that decomposes (DOC_f)	Carbon that is ultimately degraded and released to the atmosphere as CO_2 or CH_4. Average value for solid waste materials.	0.5	IPCC (2006)
	Specific value for wood, based on the value for experimental decay of branches.	**0.23**	NIR (2012)<br>Barlaz (1998).<br>Lippke et al. (2011)
	Paper	**0.49**	
	Data derived from laboratory decomposition experiments for a range of timbers	0.081 – 0.157	Wang et al. (2011)
Decay rate of wood products in landfill	Default value for wood in wet temperate climate	k = 0.03 yrs^{-1}	IPCC (2006)
	Derived from experimental decomposition of a range of timbers (range of 0 – 0.2, mean = 0.05) and assuming this occurs over 50 years.	k = 0.001 yr^{-1}	Wang et al. (2011)
	Field measurement of 18% carbon loss in hardwood products in landfill after 46 years, and assuming exponential decay.	**k = 0.004 yr^{-1}**	Ximenes et al. (2008b)
	Annual decay rate derived from quantity of landfill, methane generated and carbon remaining in landfill	k = 0.002 yr^{-1}<br>k = 0.033 yr^{-1}	Richards et al. (2007)<br>Micales and Skog (1997)
	Average values for the range of products in landfills	k=0.02–0.07 yr^{-1}<br>k = 0.02 yr^{-1}	Barlaz (2004)<br>Lippke et al. (2011)
Decay rate of paper products in landfills		**k = 0.05 yr^{-1}**	NIR (2012)
Carbon storage factor	Fraction of the carbon in the original product that remains in the landfill	0.57	Barlaz 2004

S2.2. Mountain Ash forest in the Central Highlands of Victoria

S2.2.1 Biomass carbon stock in the forest

Current carbon stock was estimated spatially across the region based on measurements of biomass at 54 research sites and estimates of biomass from 876 State Forests' inventory sites of different ages (Keith et al. 2014a). Biomass measurements included all components of living and dead biomass, except that a default root : shoot ratio of 0.2 for eucalypt forests (Snowdon et al. 2000) was used to estimate belowground biomass. Biomass was related to dominant tree age at the site. Spatial distribution of forest age across the region was determined from maps of logging and fire history. These site-based estimates of carbon stocks were scaled up across the region by accounting for landscape variability at a resolution of 250 m using the statistical modelling approach of Keith et al. (2010) that related carbon stock to environmental variables and disturbance history.

S2.2.2 Biomass accumulation rate in the forest

The biomass accumulation rate in even-aged regeneration was derived from site data relating carbon stock to forest age from various sources, including measured sites and estimates from inventory sites in the current study, as well as site data and equations from the literature (Keith et al. 2014a). In deriving the relationship, we used only those sites where time since disturbance reflected predominant age of the trees (Fig C).

Total living biomass (tC ha^{-1}) = 1200 * (1 – exp(-0.0045 * *age*)) ^0.7 (S2-3)

where, total living biomass is above and belowground, and *age* is the predominant age in years of the trees at the site, n = 119, $r^2 = 0.72$. The greatest tree age at measured sites was approximately 250 years, which had an average total living biomass of 930 tC ha^{-1}. This was not necessarily the maximum biomass for the forest type.

An additional equation to describe the biomass accumulation rate was applied in the model of carbon stock dynamics and tested in the sensitivity analysis. Grierson et al. (1992) derived a growth equation using state forest inventory data with a maximum forest age of 76 years. The original form of the equation was a polynomial and so could not be used to estimate forests older than the calibration data. To compare equations over the time periods of mature and old growth forests, we converted this equation to the form of the Chapman-Richards equation, like Equation (S2-3).

Aboveground living biomass (tC ha^{-1}) = 620 * (1 – exp(0.0065 * *age*)) ^0.75 (S2-4)

S2 Fig C. Accumulation of carbon stock in living above-and below-ground biomass in regrowth Mountain Ash forest.

Biomass at research sites and inventory sites was calculated in the current study, data from the literature included Ashton (1976), Feller (1980) and Polglase and Attiwill (1992).

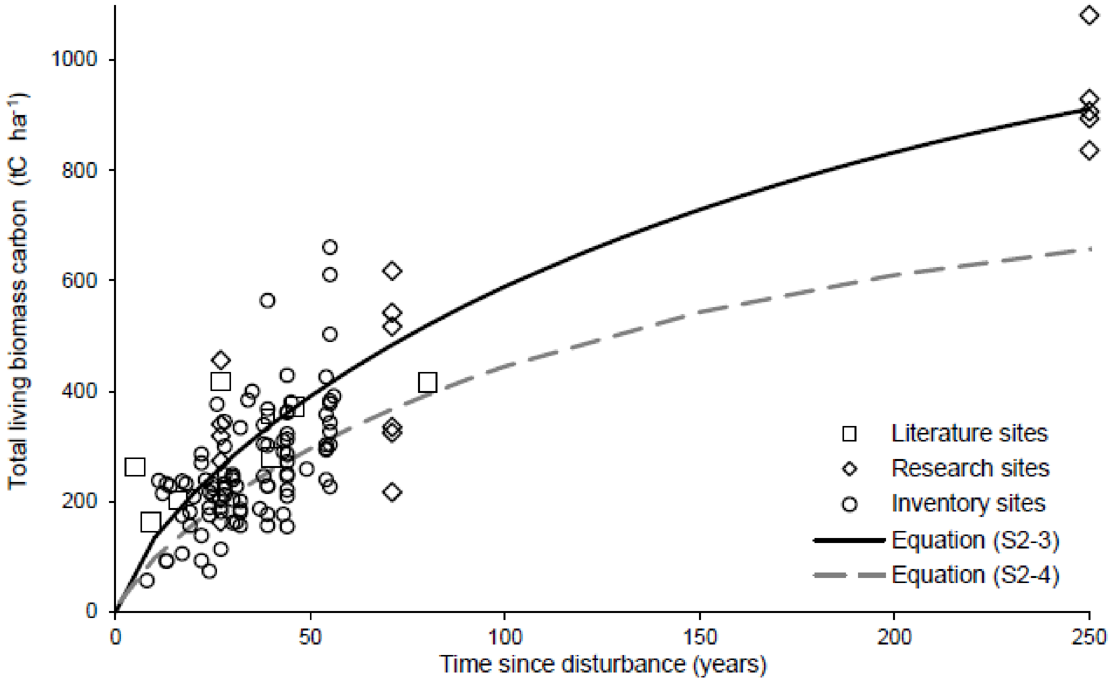

S2.2.3 Simulation of carbon stock change in harvested forests

Regrowth of Mountain Ash forest is mostly even-aged following clearfelling and slash burning, or wildfire and salvage logging. Maximum mean annual increment of the regrowth is achieved by approximately 50 years (Flint and Fagg 2007). The time of maximum increment is usually the most efficient time for harvesting in production forests. The rotation length in Mountain Ash forests is nominally 80 years (Flint and Fagg 2007, Flinn et al. 2007). However description of the historical management of these forests states that by the mid-1980s the wider spaced 1939 regrowth had reached harvestable age (Lutze et al. 1999), by the late 1980s full scale harvesting commenced, and by the mid-2000s regrowth forests supplied 80% of the State's ash timber resource (Flinn et al. 2007).

The age of forest at which it was harvested was analysed from spatial logging and fire history data (LastLog25 2011, VicForests, and FireHistory100 2011, DSE). Analysis was restricted to areas of regrowth Mountain Ash after wildfire (mostly 1939). Salvage logging was not included as it would have negatively distorted length of cutting cycle calculations, and hence the decade following wildfire was excluded from the analysis. Data for salvage logging were available following the 2007 and 2009 wildfires and so these areas were explicitly attributed and shown graphically. All silvicultural treatments except thinning from below were included. The maximum possible age in this analysis was 72 years, based on data from 1949 to 2011. The total area where forest age could be determined was 72,982 ha and the area where age at time of logging was determined was 38,135 ha. An area of

13,680 ha was unlogged but burnt in wildfire. The remaining area available for logging in 2011 was 21,167 ha

The distribution of area logged over time is shown in Fig D. The median age for harvesting is 68 years and the 25th percentile is 50 years. Hence, more than half the available area of 1939 regrowth had been logged by 2011, and before the nominal rotation length of 80 years. Rotation lengths of 50 to 80 years were tested in the sensitivity analysis to cover the range in possible forest ages.

The time over which the remaining area of regrowth could be logged depends on the logging intensity. The proposed area for logging is determined by the Timber Release Plans (TRP) over the period 2011 - 2016 (DSE 2012), although this area is not necessarily all logged (as advised by VicForests). The proposed area for logging is an average of 1656 ha yr^{-1} and 732 ha yr^{-1} of salvage logging over the 5 year period. We tested a range of intensity factors for the proposed logging area to determine the range in time over which the current available regrowth could be logged. A low intensity used a factor of 0.3 of the TRP area (as advised by VicForests) to calculate the annual area of future logging, which resulted in the most conservative estimate of 40 years. This low intensity rate of logging is similar to the actual logging rates reported for 2011 – 2014, although salvage logging rates were higher post-fire in 2009-10. A medium intensity of future logging was estimated from the rate of logging in the last year pre-2007 wildfire, which resulted in an estimate of 29 years. A high intensity of future logging at the stated rate of the TRPs results in an estimate of 10 years of logging in the available forest area.

S2 Fig D. Area of 1939 regrowth forest logged over time as area per year and cumulative area.

Forest age starts at 12 years to exclude post-fire salvage logging of unknown age. Areas logged are from 1949 to 2011, and salvage logging after the 2007 and 2009 fires (red arrows). The proposed area of logging is shown as a range of three potential logging intensities based on the Timber Release Plans. The 25th percentile of area logged is 50 years and the median is 68 years.

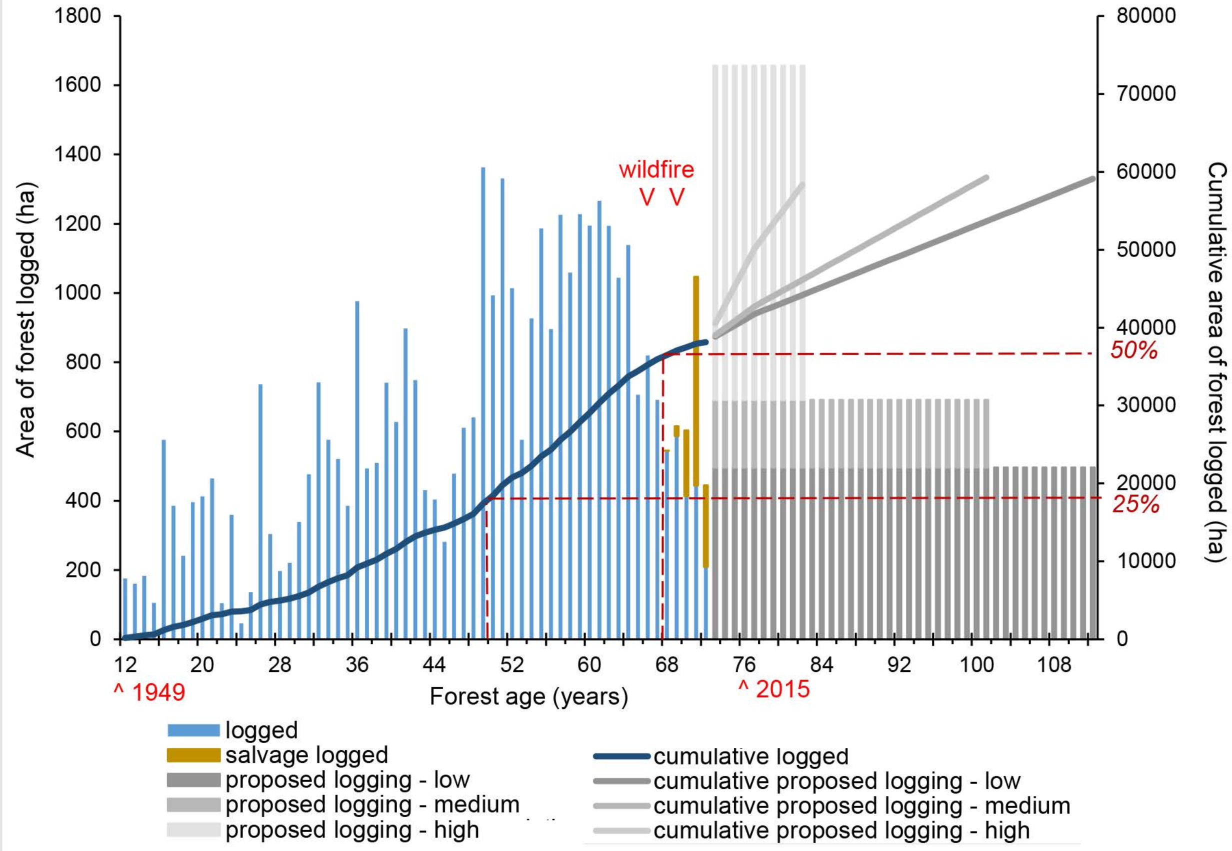

Changes in carbon stocks were analysed at the site scale of even-aged regrowth and at the regional scale accounting for the spatial distribution of age classes of forest and environmental variability across the landscape. Forest age was determined from spatial data of logging and fire history, where regeneration time was defined from selected stand-replacing disturbance events.

S2.2.4 Harvested wood products

Transfers of biomass carbon stocks in the harvested Mountain Ash forest system were derived from sources of data in the literature, particularly from NCAS and Victorian State Forests (DSE 2009, NIR 2012, Keith et al. 2014a). From the total biomass carbon stock in the forest, 0.4 is removed off-site as merchantable wood, which is transferred to 0.04 as sawn timber products and 0.2 as paper products (Fig E). The 0.6 of biomass remaining on-site as slash is burnt, with half the carbon stock combusted and the other half remaining (Gould and Cheney 2007), where it decomposes at a rate of 0.0486 yr^{-1}, based on estimates from coarse woody debris (Mackensen and Bauhus 1999). The allocation of different wood products, their longevities, and the proportion transferred to landfill, are based on the

values from the NCAS (NIR 2012) and State Forests' harvesting reports (DSE 2009) (Table F, with more details and comparisons with other studies in Keith et al. 2014a).

S2 Fig E. Transfers of carbon stocks in a harvested forest of Mountain Ash in the Central Highlands of Victoria.

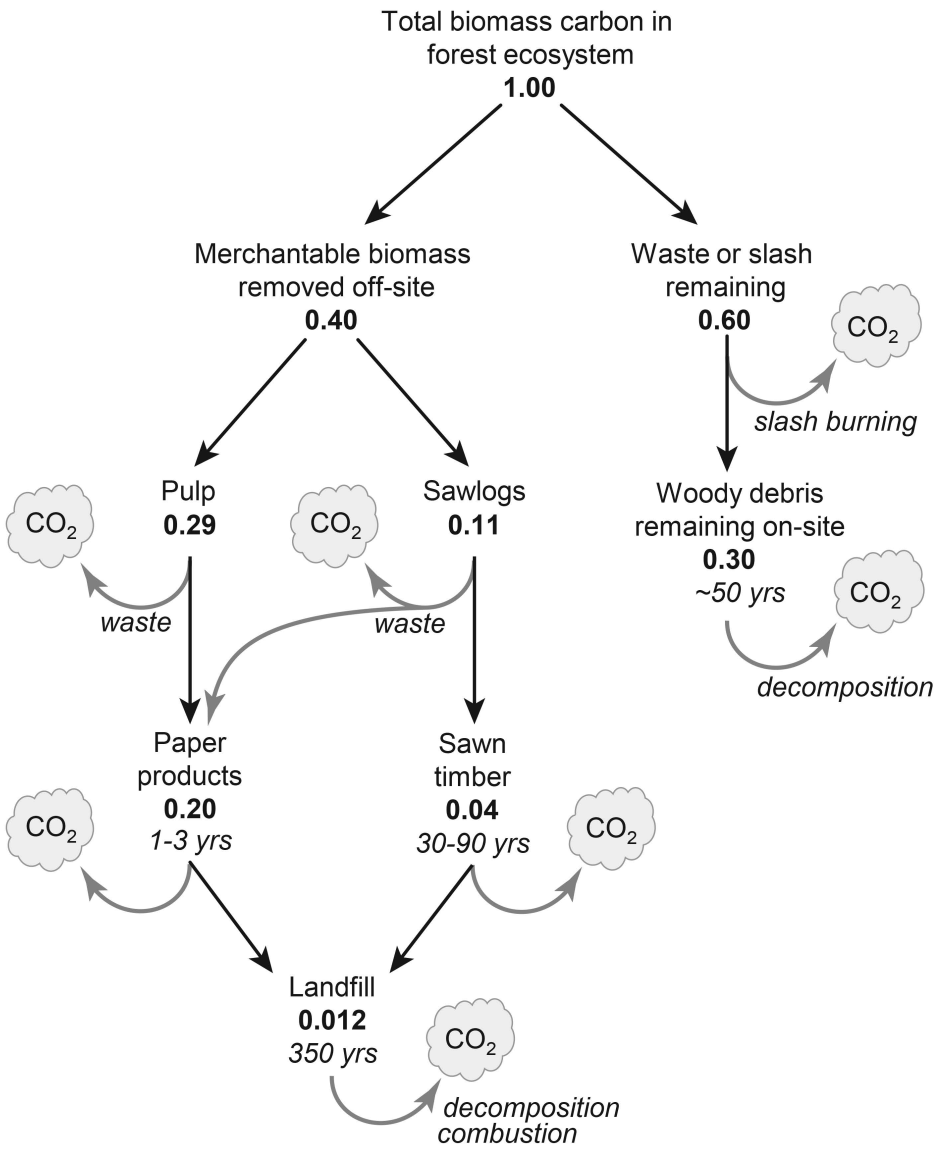

S2 Table F. Transfer of biomass carbon stocks during the harvesting process.

Values in bold were used in the simulation.

TB total biomass, LB living biomass, AGB aboveground biomass

Biomass transferred	Longevity $t_{0.95}$ (yrs)	Derivation of estimate	Ref
**Biomass removed off-site**			
**40% of TB**		Default value for moist, high quality forest under integrated harvesting for sawlog and pulpwood used in NCAS	1
20 – 80% of TB		Range for eucalypt forests used in NCAS	1
44% of TB		Site data from ash forests. Range in biomass remaining as slash of 42 – 102 tC ha^{-1} but up to 275 tC ha^{-1}.	2
45% of TB		Estimated average for eucalypt forests in Victoria	3
26% of AGB<br>21% of LB		Site data for *E. regnans* with temperate rainforest understorey in Tasmania.	4
58% of AGB<br>46% of LB		Site data for tall, wet regrowth forest of *Corymbia maculata* on the south coast of NSW.	5,6,7
70% of AGB<br>56% of LB		Site data for tall, wet regrowth forest of *E. obliqua* on the south coast of NSW.	5,6,7
45% of AGB<br>36% of LB		Site data for tall, wet regrowth forest of *E. pilularis* on the south coast of NSW.	5,6,7
37% of TB		Average biomass carbon density in areas proposed for logging = 409 tC ha^{-1}<br>Wood product harvested from clearfelling = 600 m^3 ha^{-1} = 150 tC ha^{-1} (where wood density = 0.5 t m^{-3}, carbon concentration = 0.5 g g^{-1})	8,9
50–77% AGB<br>40–62% of LB		Estimated range in expansion factors for proportion of aboveground biomass used for wood products.	10
45 – 65% AGB		Site measurements for different eucalypt species in regrowth forests in NSW	33
**Combusted in slash burning**			
**50% of slash**<br>**30% of TB**		Average burning efficiency including all available fuel present (slash, understorey, fallen dead trees, CWD, litter) over the total area combusted.	11
50% of slash		50% for CWD (> 100 mm log sizes having 80% loss over 70% of coupe area for 90% of coupes), and 70% for fine fuels (< 100 mm log sizes having 100% loss over 80% of coupe area for 90% of coupes).	2
58–63% of slash		Site data from tall, wet *E. obliqua* forest in Tasmania	12
31–89% of slash		Site data from tall, wet *E. diversicolor* forest in Western Australia.	13
**CWD remaining on-site**			

**50% of slash<br>30% of TB**	**43**	CWD from *E. regnans* for logs 10 – 30 cm diameter of the ground surface	14,15
	92	Average for Australian timber species	14
	38	Average decomposition of CWD in temperate deciduous forests.	16
	28 - 91	Chronosequence and time series site data for boreal forests	17
	10	IPCC default value for decomposition of all litter	31
**Dead coarse roots**			
	214	Estimated half-life ($t_{0.5}$) of 50 years in moist eucalypt forest	19
**Sawlogs**			
**28% of roundwood<br>11% of TB**		Harvesting reports of products from merchantable roundwood for the forest management area	9
50% of roundwood		Average value in a sawlog-driven industry	20,21<br>22
**Pulpwood**			
**72% of roundwood<br>29% of TB**		Harvesting reports of products from merchantable roundwood for the forest management area	9
**Sawn timber products**			
**33% of sawlogs<br>4% of TB**	**30 - 90**	Timber products including sawn timber, plywood and veneer.	23,24<br>25,26<br>27,28
	30 - 300	Range of lifetimes for sawn timber products used in the USA	29
	100	Global carbon budget default value for lifetime for timber products	30
	152	IPCC default value for lifetime for timber products	31
	169	Timber products from the Pacific Northwest	20
**Paper products**			
**66% of pulpwood<br>20% of TB**	**1 - 3**	Paper products including 60% in packaging, cardboard, newsprint, household and sanitary paper and 40% in printing and writing paper with lifetimes of 1 – 3 years.	24,25<br>26,32
	10	Global carbon budget default value for lifetime for pulp and paper products	30
	9	IPCC default value for lifetime for paper products	31
**Landfill**			
	152<br>(100–300)	IPCC default value for the lowest rate of decay (boreal and temperate, dry, wood waste)	34
50% of waste decomposes		Assumed anaerobic conditions and the organic material includes lignin	34

Sources are: 1, Raison and Squire (2007); 2, Flinn et al. (2007); 3, Noriss et al. (2010); 4, Green (2002); 5, Ximenes et al. (2004); 6, Ximenes et al. (2008a); 7, Ximenes and Gardner (2005); 8, Current study; 9, DSE (2009); 10, Snowdon et al. (2000); 11, Gould and Cheney (2007); 12, Slijepcevic (2001); 13, McCaw et al. (1997); 14, Mackensen et al. (2003); 15, Mackensen and Bauhus (2003); 16, Harmon (2002); 17, Harmon et al. (2000); 18, IPCC (2006); 19, Ximenes and Gardner (2006); 20, Harmon et al. (1996); 21, Harmon (2001); 22, Perez-Garcia et al. (2005); 23, Skog and Nicholson (1998); 24, Jaako Pöyry (1999); 25, Jaako Pöyry (2000); 26, Richards et al. (2007); 27, Ximenes et al. (2004); 28, Ximenes et al. 2008c; 29, Krankina nd Harmon (2006); 30, Le Quéré et al. (2012); 31, IPCC (2006); 32, Australian Paper Industry (2004); 33, Ximenes et al. (2008a); 34, Pipatti et al. (2006).

S2.3. Substitution

S2.3.1 Substitution of energy

The mitigation benefit of substitution depends on the energy output per unit of carbon emissions, which is defined as the displacement factor for energy. Calculation of the displacement factor for use of bioenergy assumes sustainable production of biomass, accounts for the type of fossil fuel and the efficiency of its substitution (Schlamadinger et al. 1995). However, the factor also includes emissions during the supply chain of bioenergy production, such as transport (FAO 2010) and waste processing (Marland and Marland 1992). Hence, use of bioenergy is unlikely to be carbon neutral (Johnson 2009). Biomass has a lower energy content compared to fossil fuels. Therefore, a larger volume and mass of material is required to generate the same energy, and so transport emissions are greater.

The displacement factor for fossil fuel energy (D_f) is defined as:

$$D_f = (E_w / E_f) \times (F_f / F_w) \quad \text{(S2-5)}$$

where, E_w = energy efficiency of the wood bioenergy system
E_f = energy efficiency of the displaced fossil fuel system
F_f = carbon emission per Joule of fossil fuel
F_w = carbon emission per Joule of wood bioenergy

Biomass used for electric power generation has a higher carbon emission rate and lower combustion efficiency than the fossil fuels it displaces. The range in D_f is 0.5 to 1 tC avoided emission / tC wood bioenergy (Sathre and O'Connor 2010), which depends on the type of fossil fuel displaced and the relative combustion efficiencies. Specific examples are given in Table G.

In our simulations of substitution of bioenergy, maximum production of bioenergy was derived from all sources of biomass residues. A displacement factor of 0.56 tC avoided tC^{-1} in wood bioenergy was calculated from an Australian national average energy content in biomass (16.2 GJ t^{-1} dry mass, NGAF 2014), an average thermal efficiency of electricity generation (25%, Koop et al. 2010), auxiliary losses in generation (10%, Koop et al. 2010), and average NSW emissions intensity of black coal-fired electricity generation (1.01 tCO_2-e MWh^{-1}, Clean Energy Regulator 2014). Biomass residues included 30% of the slash (considered as a maximum that would maintain site sustainability), wood chips previously

used for pulp, and wood products at the end-of-life with a displacement factor of 0.47 tC tC^{-1} (Ximenes et al 2012a). A displacement factor for biomass residues of 0.8 tC tC^{-1} (Ximenes 2012a) was also used for comparison in the sensitivity analysis and for comparison with the FCNSW results.

S2 Table G. Displacement factors for using wood bioenergy instead of fossil fuel energy.

Energy source displaced	tC avoided emission / tC in wood bioenergy	Reference
coal	0.8 – 0.9	FAO (2010)
coal	0.8	Ximenes et al. (2012a)
coal	0.75	Marland and Marland (1992)
oil	0.8	Schlamadinger et al. (1995)
oil	0.97	Oliver et al. (2014)
gas	0.65	Lippke et al. (2011)
gas	0.70	Oliver et al. (2014)
fossil fuels general	0.6	Marland (1997b)

S2.3.2 Substitution of products

The mitigation benefit of using wood products instead of other materials that have a higher embodied energy depends on the energy input required and the resulting carbon emissions. This displacement factor for products (D_p) is defined as:

$$D_p = (-E_w \times F_w + E_{NW} \times F_{NW} \times M_{NW/W} \times L_{W/NW}) \times (1/C_W) \quad \text{(S2-6)}$$

where, Ew = energy required to make 1 Mg of wood products (GJ/Mg)

Fw = C emission rate for fuel used to make wood products (Mg C/GJ)

E_{NW} = energy required to make non-wood products (GJ/Mg)

F_{NW} = C emission rate for fuel used to make non-wood products (Mg C/GJ)

$M_{NW/W}$ = ratio of the mass of non-wood material to wood material to make products that provide the same service (Mg/Mg)

$L_{W/NW}$ = ratio of the mean service lifetime of products from wood and non-wood materials (yr/yr)

C_W = C content of dry wood (Mg C/Mg wood)

This displacement factor includes only the direct saving in fossil fuels used for production. The range in displacement factors is 0.5 to 1 tC avoided emission tC^{-1} wood product, with higher factors used for long-lived products and high substitution efficiency (Schlamadinger and Marland 1996b). Wood products have low embodied energy, that is, the energy used in the forestry production process (management, harvesting, transport, regeneration) is on average 6% of the energy content of the logs harvested from native forests (Lippke et al. 2011; May et al. 2012).

The displacement factor is greater if all greenhouse gas emission reductions are incorporated, including fossil fuel emissions from material production, process emissions, fossil fuel emissions avoided due to bioenergy generation from biomass by-products and

post-use products, carbon storage in forests, wood products and landfill, and emissions from landfill. There is high uncertainty about which of these emissions reductions should be included in the substitution and which should be accounted separately. Hence, there is the likelihood of double counting some emission reductions. A range of displacement factors for different construction materials is given in Table H. An average factor of 2.1 tC avoided emission tC^{-1} wood product was estimated by Sathre and O'Connor (2010) from studies that incorporated all emissions reductions. In out simulations, we used this average displacement factor, but separated the factor into 1 tC tC^{-1} in the wood product and 1.1 tC tC^{-1} as displacement.

S2 Table H: Displacement factors for wood products substituting for other construction materials.

Product displaced	tC avoided emissions tC^{-1} in wood product	Reference
construction materials - average	2.2	European Wood Factsheet (2014)
steel	2.0	Abbott (2008)
plastic	1.9	Abbott (2008)
roofing tiles	0.7	Abbott (2008)
steel	1.4	Lippke et al. (2011)
concrete	1.9	Lippke et al. (2011)
steel house construction	1.3	Perez-Garcia et al. (2005)
concrete flooring	2.6	Perez-Garcia et al. (2005)
long-lived products<br>short-lived products	0.5<br>0.25	Marland et al. (1997b)
average	2.0	Ryan et al. (2012)
average products (n = 21)	2.1<br>average low is 0.8,<br>average high is 4.6	Sathre and O'Connor (2010)

S2.3.3 Substitution with plantation wood products

Biomass carbon stock and stock changes in harvested pine and eucalypt plantations were calculated using the Australian government's forest carbon model, FullCAM, which is used in the National Carbon Accounting System (NCAS) (Richards and Evans 2004, NIR 2012). Carbon stocks accumulate in the growing forest and debris and wood products are produced at the end of rotation. Representative forest plots of plantations were selected from FullCam to be in similar regions to the native forest case studies in southern NSW and Victoria (Table I). Carbon stock in wood products removed off-site was apportioned into product types and waste during processing based on national average values for softwood sawmilling for pine, or pulp and paper processing for eucalypt and pine residues (NIR 2012). Longevity of each product type and the proportion transferred to landfill followed the standard values used in the NCAS harvested wood products (HWP) model (Richards et al. 2007).

The area of land required to produce an equivalent amount of wood products of sawlogs and pulp from pine and eucalypt plantations was calculated to be 16.6% of the area of the native harvested forest in the South Coast of NSW, and 25% of the area of the Mountain Ash harvested forest (Table I).

S2 Table I. Carbon stocks estimated in pine and eucalypt plantations using the FullCAM and HWP models (NIR 2010).

	**NSW**		**Victoria**	
	**pine**	**eucalypt**	**pine**	**eucalypt**
Rotation length (years)	clearfell @ 30	clearfell @ 45<br>thinning @ 20 & 35	clearfell @ 30<br>thinning @ 15 & 20	clearfell @ 25
Carbon stock at maturity (tC ha^{-1})	143	76	161	246
Average carbon stock over rotation (tC ha^{-1})	62	37	94	134
Sawlogs products in-service (tC ha^{-1} yr^{-1})	1.26		1.58	
Pulp products in-service (tC ha^{-1} yr^{-1})	0.5	2.12	0.62	4.86
Area required for substitution of sawlogs (%)	4.6		10	
Area required for substitution of pulp (%)		12		15
