# Supplementary material for: Under What Circumstances Do Wood Products from Native Forests Benefit Climate Change Mitigation?
Source: PLoS One. 2015 Oct 5;10(10):e0139640. doi: 10.1371/journal.pone.0139640 (PMC4593608; doi:10.1371/journal.pone.0139640)
Supplement: S3 Appendix — (PDF) [file pone.0139640.s003.pdf]

S3 Appendix. Results

S3.1. Simulation of forest management scenarios

S3 Table A. Simulated carbon stock changes in case study forest types compared with reported productivity for the species.

Parameter	South Coast NSW	Mountain Ash Victoria
Mature forest carbon stock (tC ha^{-1})	115	516
Mean annual increment (tC ha^{-1} yr^{-1})	1.64	6.45
MAI for species (m^3 $ha^{-1}yr^{-1}$)	4.1 #	14 *
Increment converted from MAI (tC ha^{-1} yr^{-1})	1.44	5.9
Biomass removed off-site (tC ha^{-1} yr^{-1})	0.36	1.58

References: # Furrer (1971) in Borough (1984); * Webb (1969) in Borough (1984)

S3.1.1 Mixed native eucalypt forest on the South Coast of NSW

The simulation of changes in carbon stocks under current native forest management (reference scenario) was calculated at the local scale for the logged area on a rotation of 70 years (Fig A(A)) and for the net harvested area on a return time of 20 years (Fig A(B)). Identifying the carbon stocks in each type of harvested area in this way provided an understanding of the dynamics over time and the mosaic of areas that was aggregated at the regional scale.

S3 Fig A. Carbon stocks simulated over 100 years in selectively harvested mixed native eucalypt forest on the NSW South Coast at the local scale for (a) the area logged at a site scale on a 70 year rotation, and (b) net harvested area with a 20 year return time. Biomass in regenerated forest included living biomass above- and below-ground. Wood products represented the proportion of harvested wood products that was retained as wood products in-service. Total biomass included all pools in the harvested forest system, both on- and off-site.

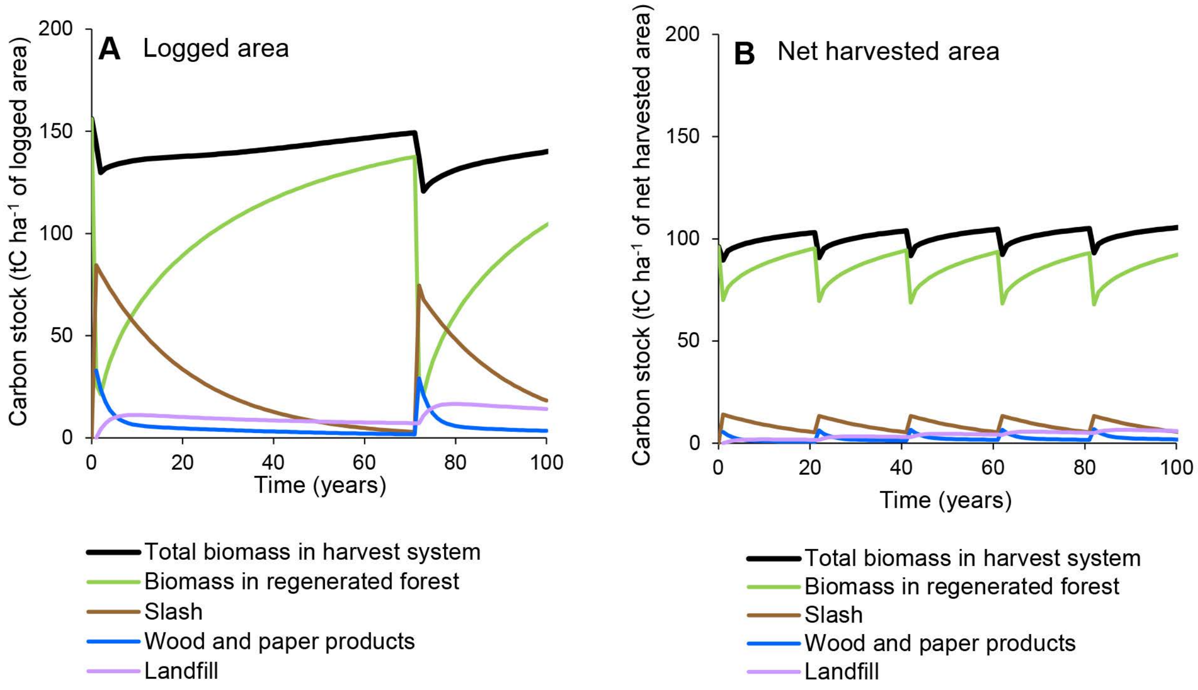

S3 Fig B. Regional average carbon stocks simulated over 100 years for the reference case and four scenarios of forest management in NSW South Coast mixed native eucalypt forest. Comparison with modelled outputs from FCNSW that were based on projected forest growth from inventory data (Ximenes et al. 2012b) (dotted lines).

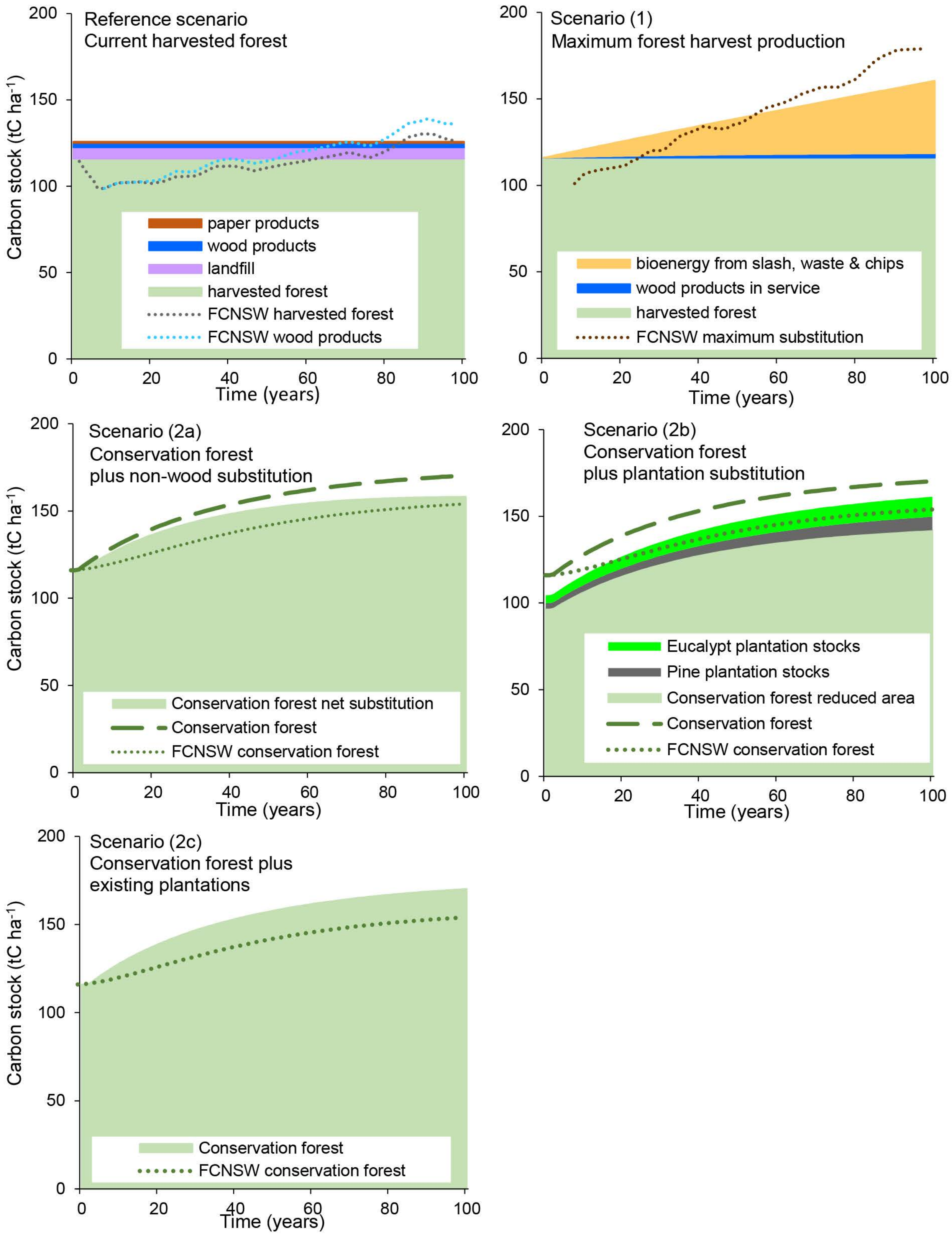

Simulated carbon stocks up to 50 years were higher in the conservation forest scenarios (Scenarios 2a, 2b, 2c) than the FCNSW model predictions. After 100 years, the FCNSW model predicted a higher carbon stock than the conservation forest scenarios that were calculated with Equation (S2-1), because of the low assumed maximum biomass value. However, when the conservation scenarios were calculated with Equation (S2-2), which was derived from site data, carbon stocks were higher at all times than the FCNSW predictions.

S3.1.2 Mountain Ash forest in the Central Highlands of Victoria

S3 Fig C. Carbon stocks simulated over 100 years at the site scale for a logged area clearfelled on an 80 year rotation in Mountain Ash forest in Victoria.
Biomass in regenerated forest included living biomass above- and below-ground. Wood products represented the proportion of harvested wood products that was retained as wood products in-service. Total biomass included all pools in the harvested forest system, both on- and off-site.

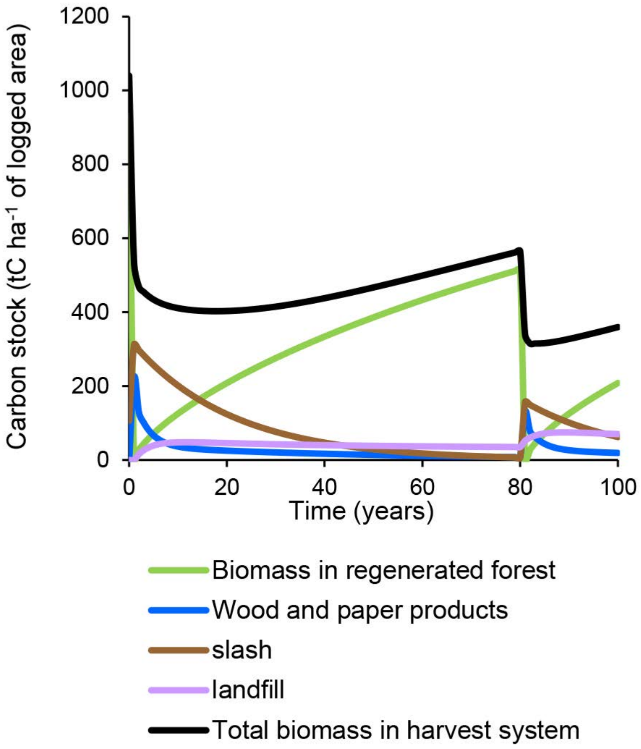

S3 Fig D. Regional average carbon stocks simulated over 100 years for reference case and four scenarios of forest management in Mountain Ash forest in Victoria. Forest carbon accumulation rate was calculated using Equation (S2-4), compared with Fig 3 which used Equation (S2-3).

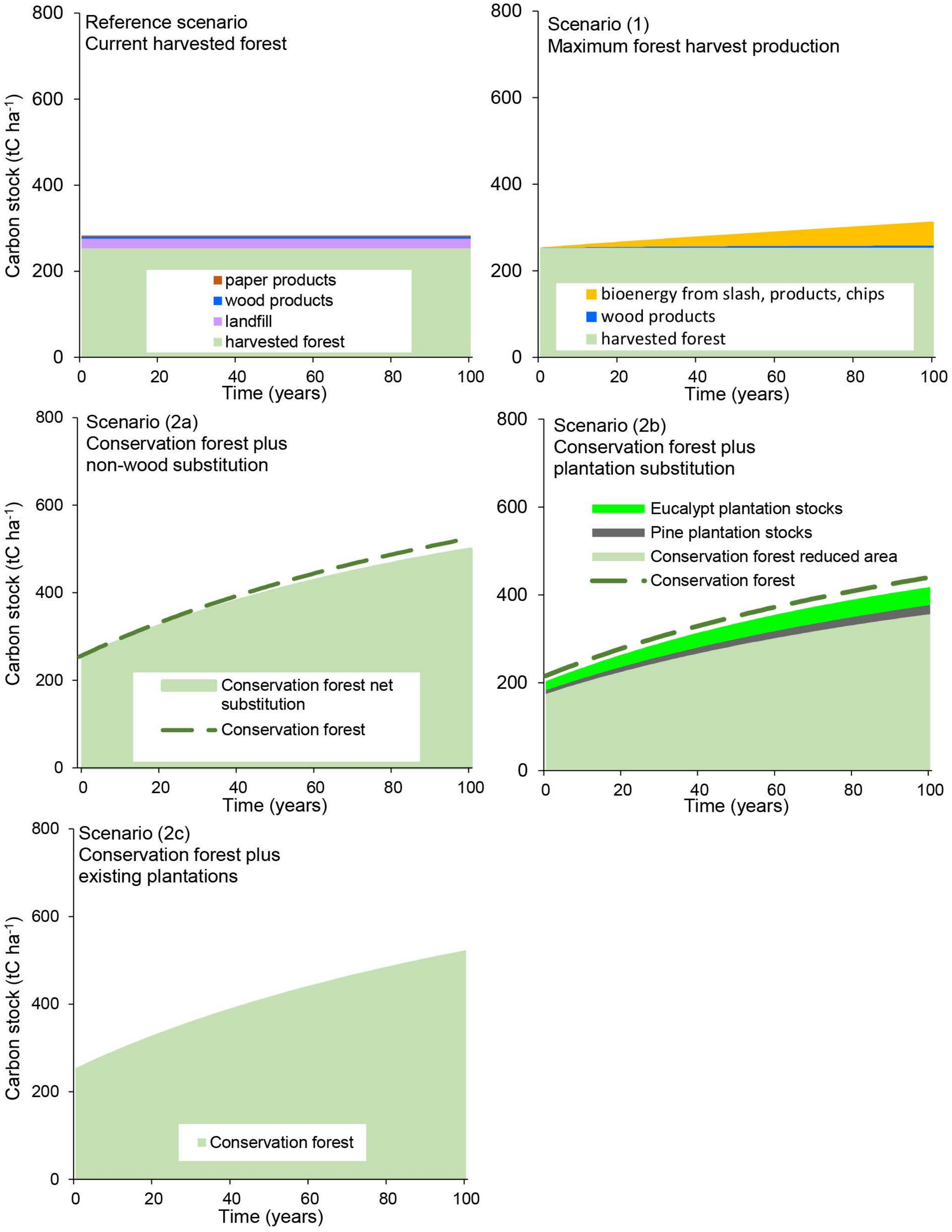

S3 Fig E. Regional average carbon stocks simulated over 100 years, including a wildfire, for the reference case and four scenarios of forest management in Mountain Ash forest in Victoria. Forest carbon accumulation rate was calculated using Equation S2-3. The forest was subject to a high severity wildfire in year 56 that killed the trees and resulted in emissions of 10% of the biomass carbon stock due to combustion. Biomass carbon stocks in the conservation forest after the fire consisted of regeneration, a small proportion of living trees, standing dead trees and coarse woody debris.

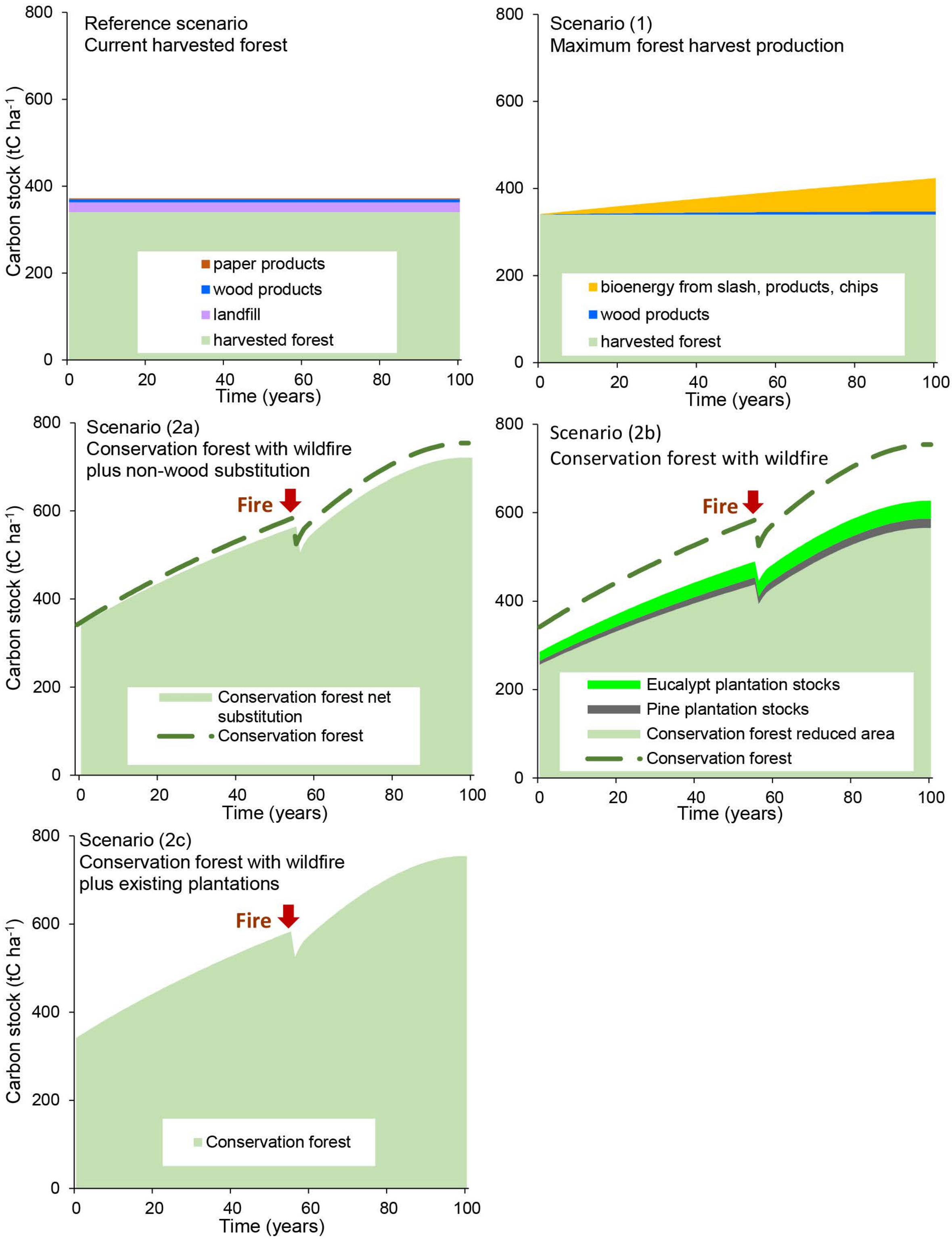

S3.2. Results of sensitivity analyses

Sensitivity analyses of the key parameters that contribute to uncertainty in the simulated carbon stock change under different forest management scenarios showed that the greatest sensitivity was due to parameters that determine the amount of biomass in the forest. In the South Coast forest, different rates of carbon accumulation predicted by Equations (S2-1) or (S2-2) resulted in a difference of 9 tC ha^{-1} after 50 years (Table B parameter (i)). In the Mountain Ash forest, different rates of carbon accumulation predicted by Equations (S2-3) or (S2-4) resulted in a difference of 148 tC ha^{-1} after 50 years (Table C parameter (i)).This difference was due to the estimated maximum potential carbon stock in a native forest, which determines the potential for sequestration of carbon in regrowing forest. In the Mountain Ash forest, the difference in carbon stock of the current forest harvesting system would be 75 tC ha^{-1} depending on the rotation length of 50 or 80 years, which influences maximum biomass accumulation in trees up to the time of harvesting (Table C parameter (xi)).

The proportion of biomass removed off-site as harvested wood products (parameter ii) varies greatly depending on forest type, silvicultural system, and the comparative measure used. These factors determine the efficiency of converting forest biomass into commercial products. In fact, increasing the proportion of harvested biomass used for products and consequently reducing the amount of slash made only a small difference to the accumulated stock, with an increase of less than 3 tC ha^{-1} over 50 or 100 years in the South Coast forest and 15.5 tC ha^{-1} in Mountain Ash forest (Tables B and C parameter (ii)). Quantifying the supply of wood products was highly uncertain and sources of information differed (Table B parameter (iii)). Differing rates of wood supply from past, present and future scenarios resulted in final stocks of carbon in wood products varying by less than 1 tC ha^{-1} over 50 years in the South Coast forest.

Information about longevities of biomass pools in wood and paper products and landfill is general and we applied the same parameter values for different forest types. Varying rates of transfers of carbon due to longevity of wood products resulted in differences in carbon stocks of 3 and 6 tC ha^{-1} after 50 and 100 years, respectively in South Coast forests (Table B parameter (iv)). Varying rates of decay in landfill and assumptions about the proportion of decomposable material resulted in a maximum difference of 1.2 tC ha^{-1} after 100 years (Table B parameter (v)).

Differentiation of wood and paper products and landfill into pools with differing longevities, based on the Harvested Wood Products (HWP) model (NIR 2012), resulted in differences in carbon stocks of 2 and 7.5 tC ha^{-1} in the South Coast forest and 5 and 9.2 tC ha^{-1} in the Mountain Ash forest after 50 and 100 years, respectively (Tables B and C parameter (vi)). Higher stocks predicted in the HWP model resulted from 0.35 of paper products having a slow decomposition rate and 0.10 not being exposed to decay, and then when transferred to landfill only 0.49 of the paper waste material was exposed to decay. When wood product

pools are differentiated, carbon in the pools with shorter longevities is transferred to landfill faster, but then remains in landfill where decomposition is very slow; irrespective of the type of product. Differentiating pools of wood products with different longevities, but not maintaining the differentiation in decomposition in landfill, could thus represent a problem in modelling transfers of carbon stocks.

The proportion of harvested wood products used for sawlogs compared with pulp influences the longevity of the carbon stocks in products, with increased amounts of sawlogs resulting in increased total carbon stock. However, these changes are relatively small because of the high proportion of product that becomes waste material (Table B parameter (ix)).

Displacement factors for substitution of products (parameter (vii)) vary depending on the product or mix of products being displaced, for example concrete, steel, aluminium, tiles, bricks, or carpet, and their embodied fossil fuel emissions. These factors are usually used as an average because there is insufficient data for case studies of wood products being directly substituted by a specific mix of non-wood products. Our results showed that the difference in carbon stock can be 4.3 or 8.6 tC ha^{-1} after 50 or 100 years, respectively. Displacement factors for substitution of bioenergy (parameter (viii)) are determined by the energy content of biomass, thermal efficiency of electricity generation from biomass, and energy requirements of the power plant, compared with the emissions from fossil fuel sources of power generation. This displacement factor is likely to decrease over time as the efficiency of bioenergy generation is improved. Hence, displacement factors can have an important effect on carbon stocks compared with other parameters related to products and energy sources, but have a lesser effect than parameters determining forest biomass.

Biomass remaining on-site post-harvest consists of the aboveground components of trees that are not commercial product such as stumps, branches, leaves, decayed wood, non-commercial species, understorey; existing coarse woody debris; and belowground biomass. An average value for the proportion of biomass that is combusted in high-intensity fires (parameter (x)) of 0.5 (Gould and Cheney 2007) was used in the base case simulation in the Mountain Ash forest. However, in a more detailed analysis, we differentiated the biomass in slash. Aboveground biomass was half combusted and half decomposed. Belowground biomass was not subject to combustion, only to decomposition. The difference in carbon stock was 13.3 or 14.4 tC ha^{-1} after 50 or 100 years, respectively. However, we did not account for the effect of the higher combustion efficiency of coarse woody debris compared with harvested green material, and the resulting additional impact of emissions of other forms of greenhouse gases, especially methane.

S3.2.1. Mixed eucalypt forest on the South Coast of NSW

S3 Table B. Simulated carbon stocks under a range of parameter values.

The first listed value for each parameter in the table was used in the base case simulation (Fig 2), and used as the base case parameter as other parameters were varied.

**Parameter values**	**Simulated carbon stock** (tC ha^{-1})			**Reference**
	20 yrs	50 yrs	100 yrs	
**(i) Biomass accumulation rate with maximum simulated carbon stock in conservation forest**				
	Forest biomass			
Eqn (B1), max. 130 tC ha^{-1}	139	158	170	Ximenes et al. (2012a)
Eqn (B2), max. 250 tC ha^{-1}	139	167	198	Current study
**(ii) Proportion of aboveground biomass removed off-site as wood products**				
	Products, landfill & slash			
0.35 (default in FullCAM)	13.0	20.4	24.5	NIR (2012)
0.61 (from commercial trees)	12.0	20.0	25.9	Ximenes et al. (2012a)
0.22 (from all trees)	14.9	22.6	26.0	Ximenes et al. (2012a)
**(iii) Wood products supply**				
	Wood products			
Proportion of forest biomass harvested: 0.275	1.65	3.16	4.33	Florence (2007)
Actual harvest volume 2005-2013: 11.8 tC/ha	1.58	3.02	4.13	IFOA (2013)
Planned harvest volume: 12.4 tC/ha	1.66	3.18	4.34	FCNSW (2013) harvest plans
Past harvesting volume: 14.6 tC/ha	1.95	3.74	5.12	Florence (2007)
Annual input to HWP model for even flow projection 2008-2057[1]	1.42	2.86		FCNSW (2009) Performance Audit Report yield forecasts
Projected wood volume to meet market supply agreement 2008-2057[2]	1.72	2.90		FCNSW (2009) Performance Audit Report yield forecasts
**(iv) Longevity of wood products**				
	Wood products			
$t_{0.5}$=35 yrs, k=0.0198 yr^{-1}	1.65	3.16	4.33	UNFCCC (2012)
$t_{0.5}$=30 yrs, k=0.0232 yr^{-1}	1.60	2.95	3.87	NIR (2012)
$t_{0.5}$=50 yrs, k=0.0139 yr^{-1}	1.74	3.59	5.37	NIR (2012)

$t_{0.5}$=90 yrs, k = 0. 0077 yr^{-1}	1.84	4.13	6.93	NIR (2012)
$t_{0.95}$=30 yrs, k = 0.10 yr^{-1}	0.87	0.99	0.99	NIR (2012)
$t_{0.95}$=50 yrs, k = 0.06 yr^{-1}	1.17	1.58	1.65	NIR (2012)
$t_{0.95}$=90 yrs, k = 0.033 yr^{-1}	1.47	2.44	2.90	NIR (2012)
**(v) Landfill decay rate**				
	Landfill stocks			
k = 0.004 yr^{-1}	0.18	0.93	2.68	Ximenes et al. (2008b)
k = 0.002 yr^{-1}	0.18	0.96	2.88	Richards et al. (2007), Ximenes et al. (2012a)
DOC_f = 0.23, k = 0.03 yr^{-1}	0.18	0.88	2.42	(NIR 2012)
DOC_f = 0.5, k = 0.03 yr^{-1}	0.17	0.78	1.88	IPCC (2006)
DOC_f = 0.12, k = 0.001 yr^{-1}	0.18	0.99	3.08	Wang et al. (2011)
**(vi) Differentiation of product and landfill pools**				
	Wood and paper products and landfill			
Current analysis	6.35	11.79	16.67	This paper
Harvested Wood Products model[3]	6.08	13.77	24.22	NIR (2012)
**(vii) Displacement factors for substitution of products**				
	Products			
1.1 tC saved / tC in wood	1.26	3.16	6.32	Displacement additional to wood product
2.1 tC saved / tC in wood	2.41	6.04	12.07	Sathre and O'Connor (2010)
2.6 tC saved / tC in wood	2.99	7.47	14.95	Perez-Garcia et al. (2005)
**(viii) Displacement factors for substitution of bioenergy**				
	Bioenergy			
0.56 tC avoided / tC in wood	6.04	14.95	29.60	Current study using national average values
0.8 tC avoided / tC in wood	8.52	21.15	42.00	Ximenes et al. (2012a)

Parameter (iii) Scenarios for wood volume projections from Forestry Corporation NSW for the South Coast forest:

[1] projection for even flow which supplies wood volumes considered to be at a sustainable yield (FCNSW 2011).

[2] wood supply agreement projections to supply wood volumes under contractual commitments to industry until 2020 under the Regional Forest Agreement, and thence reduced volumes to return to a more sustainable yield (FCNSW 2011).
[3] Parameter (vi) Harvested Wood Products model (HWP) is used in the National Carbon Accounting System (NIR 2012). Wood products are differentiated into five pools of paper and wood products, each of which pass through young, mid and old age categories with assigned longevities.

S3.2.2. Mountain Ash forest in the Central Highlands of Victoria

S3 Table C. Simulated carbon stocks under a range of parameter values.
The first listed value for each parameter in the table was used in the base case simulation (Fig 3), and used as the base case parameter as other parameters were varied.

Parameter values	Simulated carbon stock ($tC\ ha^{-1}$)			Reference
	20 yrs	50 yrs	100 yrs	
**(i) Biomass accumulation rate with maximum simulated carbon stock in conservation forest**				
	Forest biomass			
Equation (S2-3)	444	566	719	Current paper
Equation (S2-4)	330	418	523	Grierson et al. (1992)
**(ii) Proportion of total biomass removed off-site as wood products**				
	Products, landfill & slash			
0.4	27.0	43.3	55.9	Raison & Squire (2007)[#]
0.6	27.0	45.4	62.0	
0.8	27.6	49.1	71.4	
**(vi) Differentiation of product and landfill pools**				
	Wood & paper products, landfill			
Current analysis	11.2	20.8	31.7	Current paper
Harvested Wood Products model	10.1	15.9	22.5	NIR (2012)
**(ix) Proportion of HWP used for sawlogs**				
	Products, landfill & slash			
0.28	27.0	43.3	55.9	DSE (2009)
0.50	26.8	44.6	61.2	
**(x) Slash components combusted**				
	Products, landfill & slash			
0.5 of all waste material	27.0	43.3	55.9	
0.5 of AGB slash and CWD	36.4	56.6	70.3	

(xi) Rotation length				
	Total stock			
80 years	343	359	372	Flint and Fagg (2007), Flinn et al. (2007)
50 years	265	284	299	Fig D in S2 Appendix

#Value calculated from data in the appendix tables specifically for Victorian Ash forest. Parameter values with no reference given were tested in the model to provide a range of values.
