# Supplementary material for: Under What Circumstances Do Wood Products from Native Forests Benefit Climate Change Mitigation?
Source: PLoS One. 2015 Oct 5;10(10):e0139640. doi: 10.1371/journal.pone.0139640 (PMC4593608; doi:10.1371/journal.pone.0139640)
Supplement: S4 Appendix — (PDF) [file pone.0139640.s004.pdf]

S4 Appendix. References for Supporting Information

Abbott J (2008) What is a carbon footprint? Report presented to the Swedish Forest Industries Federation and Timcon by The Edinburgh Centre for Carbon Management. Ref: ECCM-EM-483-2007, Edinburgh, UK.

Ash J and Helman C (1990) Floristics and vegetation biomass of a forest catchment, Kioloa, south coastal New South Wales. Cunninghamia 2: 167 – 182.

Ashton DH (1976). The development of even-aged stand of *Eucalyptus regnans* F. Muell. in Central Victoria. Aust J Bot 24:397–414.

Australian Paper Industry (2004) Production Statistics. 2003-04. Available: http://www.a3p.asn.au/admin/assets/pdf/Stats/Statistics2003-04PaperProduction.pdf. Accessed: 14 September 2014.

Barlaz MA (1998) Carbon storage during biodegradation of municipal solid waste components in laboratory-scale landfills. Glob Biogeochem Cycles 12(2): 373 – 380.

Barlaz MA (2004) Critical review of forest products decomposition in municipal solid waste landfills. Technical Bulletin No. 872, National Council for Air and Stream Improvement, Research Triangle Park, N.C., USA.

Barlaz MA (2006) Forest products decomposition in municipal solid waste landfills. Waste Management 26: 321 – 333.

Borough CJ, Brown WE, Brown AG, Incoll WD, May JR, Bird T (1984) Yield statistics. In: Hillis WE and Brown AG, editors. Eucalypts for Wood Production. CSIRO, Academic Press, Sydney, Australia. Pp 201-228.

Clean Energy Regulator (2014) Greenhouse and energy information 2012 – 2013, Commonwealth of Australia.

DSE (2009) Department of Sustainability and Environment. Monitoring Annual Harvesting Performance in Victoria's State Forests Technical Reports for Forest Management Areas of Central, Dandenong and Central Gippsland, Victorian Government, Australia.

European Wood Factsheet (2014) No.3 Wood products as carbon stores. Available: http://www.vhn.org/pdf/Eurofact3-Wood_as_Carbon_stores.pdf. Accessed: 14 September 2014.

FAO (2010) What woodfuels can do to mitigate climate change. Food and Agriculture Organisation, Forestry Paper 162, Rome.

Farine DR, O'Connell DA, Raison RJ et al. (2012) An assessment of biomass for bioelectricity and biofuel, and for greenhouse gas emission reduction in Australia. Global Change Biology Bioenergy 4: 148-175

FCNSW (2009) Auditor General's Report Performance Audit. Sustainable Native Forest Operations – Forests NSW. Auditor General NSW Government.

FCNSW (2011) Performance Audit Report. Yield forecasts – Southern Regional Forest Agreement, South Coast sub-region. , Forestry Corporation of NSW, Sydney, Australia.

FCNSW (2013) Southern Region Harvesting Plans for each compartment in the South Coast sub-region, Forestry Corporation of NSW, Sydney, Australia.

Feller MC (1980) Biomass and nutrient distribution in two eucalypt forest ecosystems. Aust J Ecol 5: 309-333.

Flinn D, Squire R, Wareing K (2007) Victoria. In: Raison RJ and Squire R, editors. Forest management in Australia: implications for carbon budgets. National Carbon Accounting System Technical Report No. 32, Australian Greenhouse Office, Canberra, Australia. pp 103-145.

Flint A and Fagg P (2007) Mountain Ash in Victoria's State Forests. Silvicultural reference manual No. 1. Department of Sustainability and Environment, Victoria, Australia.

Florence R (1996) Ecology and Silviculture of Eucalypt Forests. CSIRO Publishing, Australia.

Florence R (2007) New South Wales. In: Raison RJ and Squire R, editors. Forest management in Australia: implications for carbon budgets. National Carbon Accounting System Technical Report No. 32. Australian Greenhouse Office, Canberra, Australia. pp 49-102.

Forster P, Ramaswamy V, Artaxo P, Berntsen T, Betts R et al. (2007) Changes in Atmospheric Constituents and in Radiative Forcing. In: Solomon S, Qin D, Manning M, Chen Z,Marquis M et al., editors. Climate Change 2007: The Physical Science Basis. Contribution of Working Group I to the Fourth Assessment Report of the Intergovernmental Panel on Climate Change. Cambridge University Press, Cambridge, United Kingdom and New York, NY, USA.

Furrer BJ (1971) Management and silviculture in the spotted gum forests on the south coast of New South Wales. MSc Thesis ANU.

Gifford RM (2000) Carbon contents of above-ground tissues of forest and woodland trees. National Carbon Accounting System Technical Report No. 22, Australian Greenhouse Office, Canberra, Australia.

Gould JS and Cheney NP (2007) Fire management in Australian forests. In: Raison RJ and Squire R, editors. Forest management in Australia: implications for carbon budgets. National Carbon Accounting System Technical Report No. 32, Australian Greenhouse Office, Canberra, Australia. pp 341-371.

Green G (2002) Esperence 74D (EPO74D) Logging coupe inventory. Timber workers for forests. Available: http://www.twff.com.au/epo74d.pdf. Accessed: 5 March 2014.

Harmon ME, Ferrell WK, Franklin JF (1990) Effects on carbon storage of conversion of old-growth forests to young forests. Science 247(4943): 699-703.

Harmon ME, Harmon JM, Ferrell WK, Brooks D (1996) Modelling carbon stocks in Oregon and Washington forest products: 1900-1992. Climatic Change 33: 521 – 550.

Harmon ME, Krankina ON, Sexton J (2000).Decomposition vectors: a new approach to estimating woody detritus decomposition dynamics. Can J For Res 30: 76–84

Harmon ME (2001) Carbon sequestration in forests: addressing the scale question. J For 99, 24 – 29.

Harmon ME (2002) Moving towards a new paradigm for woody detritus management. USDA Forest Service Gen. Tech. Rep. PSW-GTR-181.

IFOA (2013) Integrated Forestry Operations Approval for Southern Region, Forestry Corporation of NSW, NSW Government, Sydney, Australia.

Illic J, Boland D McDonald M, Downes G, Blakemore P (2000) Woody density phase 1 – state of knowledge. National Carbon Accounting System Technical Report No. 18, Australian Greenhouse Office, Canberra, Australia.

IPCC (2006) Guidelines for National Greenhouse Gas Inventories. Eggleston S, Buendia L, Miwa K, Ngara T, Tanabe K, editors. Institute for Global Environmental Strategies, Hayama, Japan. Available: http://www.ipcc-nggip.iges.or.jp/public/2006gl/index.html. Accessed 5 March 2014.

Jaakko Pöyry Consulting (1999) Usage and lifecycle of wood products. National Carbon Accounting System, Technical Report No. 8, Australian Greenhouse Office, Commonwealth of Australia, Canberra

Jaakko Pöyry Consulting (2000) Analysis of wood product accounting options for the nation accounting system. National Carbon Accounting System, Technical Report No. 24, Australian Greenhouse Office, Commonwealth of Australia, Canberra.

Janisch JE and Harmon ME (2002) Successional changes in live and dead wood carbon stores: implications for net ecosystem productivity. Tree Physiol 22: 77–89.

Johnson E (2009) Goodbye to carbon neutral: getting biomass footprints right. Environmental Impact Assessment Review 29: 165 – 168.

Jones L (1998) Carbon storage in terrestrial ecosystems: a case study in the Kioloa State Forest. BSc Hons. Dept Geography, ANU.

Keith H, Mackey BG, Berry S, Lindenmayer DB, Gibbons P (2010) Estimating carbon carrying capacity in natural forest ecosystems across heterogeneous landscapes: addressing sources of error. Glob Change Biol 16:2971-2989.

Keith H, Lindenmayer DB, Mackey B, Blair D, Carter L, et al. (2014a) Managing temperate forests for carbon storage: impacts of logging versus forest protection on carbon stocks. Ecosphere 5(6), 75: 1 – 34.

Kirschbaum MUF (2003) To sink or burn? A discussion of the potential contributions of forests to greenhouse gas balances through storing carbon or providing biofuels. Biomass Bioenergy 24: 297 – 310.

Koop K, Koper M, Bijsma R, Wonink S, Ouwens JD (2010) Evalustion of improvements in end-conversion efficiency for bioenergy production. Ecofys Netherlands. Report to the Directorate General for Energy and Transport in the European Commission, Brussels, TREN/A2/143-2007. Available: http://europa.eu.int/comm/dgs/energy_transport/forum/index_en.htm. Accessed: 5 March 2014.

Krankina ON, Harmon ME (2006) Forest management strategies for carbon storage. Pages 79 – 92 In: Cloughsey M, editor. Forests, Carbon and Climate Change: A Synthesis of Science Findings. Oregon Forest Research Institute, Portland, Oregon, USA.

LeQuérè Q, Andres RJ, Boden T, et al. (2012) The global carbon budget 1959 – 2011. Earth System Science Data Discussions 5: 1107 – 1157.

Lippke B et al. (2011) Life cycle impacts of forest management and wood utilization on carbon mitigation: knowns and unknowns. Carbon Management 2: 303–333.

Lutze MT, Campbell RG, Fagg PC (1999) Development of silviculture in the native State forests of Victoria. Aust For 62(3): 236-244.

Mackensen J, Bauhus J (1999) The decay of coarse woody debris. National Carbon Accounting System Technical Report No. 6. Australian Greenhouse Office, Canberra, Australia.

Mackensen J, Bauhus J (2003) Density loss and respiration rates in coarse woody debris of *Pinus radiata, Eucalyptus regnans* and *Eucalyptus maculata*. Soil Biol Biochem 35: 177 – 186.

Mackensen J, Bauhus J, Webber E (2003) Decomposition rates of coarse woody debris – A review with particular emphasis on Australian tree species. Aust J Bot 51: 27 – 37.

Marland G, Marland S (1992) Should we store carbon in trees? Water, Air and Soil Pollution 64: 181 – 195.

Marland G, Schlamaginger B (1995) Biomass fuels and forest management strategies: How do we calculate the greenhouse gas emissions benefits? Energy 20(11): 1131 – 1140.

Marland G, Schlamaginger B (1997) Forests for carbon sequestration of fossil fuel substitution? A sensitivity analysis. Biomass Bioenergy 13(6): 389 – 397.

Marland G, Schlamadinger B, Canella L (1997a) Forest management for mitigation of CO_2 emissions: How much mitigation and who gets the credits? Mitigation and Adaptation Strategies for Global Change 2: 303 – 318.

Marland G, Schlamadinger B, Leiby P (1997b) Forest biomass based mitigation strategies: Does the timing of carbon reductions matter? Critical Reviews in Environmental Science and Technology 27: S213 – S226.

May B, England JR, Raison RJ, Paul KI (2012) Cradle-to-gate inventory of wood production from Australian softwood plantations and native hardwood forests: embodied energy, water use and other inputs. For Ecol Manage 264: 37 – 50.

McCaw WL, Smith RH, Neal JE (1997) Prescribed burning of thinning slash in regrowth stands of karri (*Eucalyptus diversicolor*). 1. Fire characteristics, fuel consumption and tree damage. International J Wildland Fire 7(1): 29 – 40.

Micales JA, Skog KE (1997) The decomposition of forest products in landfills. International Biodeterioration and Biodegradation 39(2-3): 145 – 158.

NIR (2010) National Inventory Report 2012. The Australian Government submission to the United Nations Framework Convention on Climate Change. Australian National Greenhouse Accounts. Department of Environment, Australian Government, Canberra.

NIR (2012) National Inventory Report 2012. The Australian Government submission to the United Nations Framework Convention on Climate Change. Australian National Greenhouse Accounts. Department of Environment, Australian Government, Canberra.

Norriss J, Arnold S, Fairman T (2010) An indicative estimate of carbon stocks on Victoria's publicly managed land using the FullCAM carbon accounting model. Aust For 73: 209 – 219.

Oliver CD, Nassar NT, Lippke BR, McCarter JB (2014) Carbon , fossil fuel, and biodiversity mitigation with wood and forests. J Sustainable For 33: 248-275.

Perez-Garcia J, Lippke B, Comnick J, Manriquez C (2005) An assessment of carbon pools, storage, and wood product market substitution using life-cycle analysis results. Wood and Fiber Science37: 140-148.

Pipatti R, Sharma C, Yamada M, et al. (2006) Waste generation, composition and management data. In: IPCC Guidelines for National Greenhouse Gas Inventories, Vol. 5 Waste, Chapter 2.

Polglase PJ, Attiwill PM (1992) Nitrogen and phosphorus cycling in relation to stand age of *Eucalyptus regnans* F.Muell. Pl Soil 142: 157 – 166.

Raison RJ, Squire RO (2007) Forest management in Australia: implications for carbon budgets. National Carbon Accounting System Technical Report No. 32. Australian Greenhouse Office, Canberra, Australia.

Reid H, Huq S, Inkinen A, MacGregor J, Macqueen D et al. (2004) Using wood products to mitigate climate change: a review of evidence and key issues for sustainable development. Edinburgh Centre for Carbon Management, Edinburgh, UK.

Richards F (1959) A flexible growth function for empirical use. J Exper Bot 10: 290 – 300.

Richards G, Evans D (2004) Development of a carbon accounting model (FullCAM vers. 1.0) for the Australian continent. Aust For 67, 277–283.

Richards G et al. (2007). Developing a carbon stocks and flows model for Australian wood products. Aust For 70, 108–119

Righelato R, Spracklen DV (2007) Carbon mitigation by biofuels or by saving and restoring forests? Science 317: 902

Roxburgh SH, Wood SW, Mackey BG, Woldendorp G, Gibbons P (2006) Assessing the carbon sequestration potential of managed forests: a case study from temperate Australia. J Appl Ecol 43: 1149 – 1159.

Ryan MG, Harmon ME, Birdsey RA, Giardina CP, Heath LS, et al. (2012) A synthesis of the science on forests and carbon for US forests. Issues in Ecology 13: 1-11.

Sathre R, O'Connor J (2010) Meta-analysis of greenhouse gas displacement factors of wood product substitution. Environ Sci Policy 13: 104 – 114.

Schlamadinger B, Spitzer J, Kohlmaier GH, Lüdeke M (1995) Carbon balance of bioenergy from logging residues. Biomass and Bioenergy 8(4): 221 – 234.

Sclamadinger B, Marland G (1996a) Full fuel cycle carbon balances of bioenergy and forestry options. Energy Conservation Management 37(6-8): 813 – 818.

Sclamadinger B, Marland G (1996b) The role of forest and bioenergy strategies in the global carbon cycle. Biomass and Bioenergy 10(5/6): 275 – 300.

Skog, K.E. and Nicholson, G.A. (1998) Carbon cycling through wood products: the role of wood and paper products in carbon sequestration. For Prod J 48(7/8) 75 – 83.

Skog, K.E. and Nicholson, G.A. (2000) Carbon sequestration in wood and paper products. USDA Forest Service General Technical Report RMRS-GTR-59.2000, Ch 5 pp 79 – 88.

Slijepcevic A (2001) Loss of carbon during controlled regeneration burns in *Eucalyptus obliqua* forest. Tasforests 13(2): 281 – 290.

Snowdon P, Eamus D, Gibbons P, Khanna P, Keith H, et al. (2000) Synthesis of allometrics, review of root biomass and design of future woody biomass sampling strategies. NCAS Technical Report No. 17 Australian Greenhouse Office, Canberra.

Tucker SN, Tharumarajah A, May B, England J, Paul K, et al. (2009) Life cycle inventory of Australian forestry and wood products. Forest and Wood Products Australia Ltd, Project No. PNA008-0708, Melbourne, Victoria.

Turner J, Lambert M (1986) Effects of forest harvesting nutrient removals on soil nutrient reserves. Oecologia 70: 140 – 148.

Turner J, Lambert M, Holmes G (1992) Nutrient cycling in forested catchments in south-eastern New South Wales. 1. Biomass accumulation. For Ecol Manage 55: 135 – 148.

UNFCCC (2012) Report of the Conference of the Parties on its seventeenth session, Durban December 2011. FCCC/CP/2011/9

Wang X, Padgett JM, De la Cruz FB, Barlaz MA (2011) Wood biodegradation in laboratory-scale landfills. Environ SciTech 45: 6864 – 6871.

Woldendorp G (2000) Estimating carbon in mature eucalypt forests. BSc Hons thesis, Dept Geography, ANU.

Woldendorp G, Keenan RJ, Ryan MF (2002) Coarse woody debris in Australian forest ecosystems. Criteria and indicators of sustainable forest management. Report for the National Greenhouse Strategy. Module 66 Bureau of Rural Resources, Canberra, Australia.

Ximenes F, Gardner WD, Marchant JF (2004) Total biomass measurement and recovery of biomass in log products in Spotted Gum (*Corymbia maculata*) forests of SE NSW. National Carbon Accounting System, Technical Report No. 47, Australian Greenhouse Office, Canberra.

Ximenes FA, Gardner WD (2005) Recovery of biomass as green sawn boards after milling of Spotted Gum (*Corymbia maculata*) sawlogs from NSW south coast forests. National Carbon Accounting System Technical Report No. 48, Australian Greenhouse Office, Canberra.

Ximenes FA, Gardner WD (2006) The decay of coarse woody roots following harvest in a range of forest types. National Carbon Accounting System Technical Report No. 49, Australian Greenhouse Office, Canberra.

Ximenes FA, Gardner WD, Richards GP (2006) Total above-ground biomass and biomass in commercial logs following the harvest of spotted gum (*Corymbia maculata*) forests of SE NSW. Aust For 69 (3): 213 – 222.

Ximenes FA, Gardner WD, Kathuria A (2008a) Proportion of aboveground biomass in commercial logs and residues following the harvest of five commercial forest species in Australia. For Ecol Manage 256: 335 – 346.

Ximenes FA, Gardner WD, Cowie AL (2008b) The decomposition of wood products in landfills in Sydney, Australia. Waste Management 28: 2344 – 2354.

Ximenes F, Kapambwe M, Keenan R (2008c) Timber use in residential construction and demolition. BDP Environmental Design Guide, PRO 36: 1 – 8.

Ximenes F, George B, Cowie A, Kelly G, Williams J, et al. (2012a) Harvested forests provide the greatest ongoing greenhouse gas benefits. Does current Australian policy support optimal greenhouse gas mitigation outcomes? Department of Primary Industries, NSW Government, 60 pp.

Ximenes FA, George B, Cowie A, Williams J, Kelly G (2012b) Greenhouse gas balance of native forests in New South Wales, Australia. Forests 3: 653 – 683.
